# Rational Formulation of Ether‐Lactone Electrolytes for Safe and Sustainable Ni‐Rich Lithium‐Ion Batteries

**DOI:** 10.1002/anie.202526145

**Published:** 2026-04-24

**Authors:** Juan Luis Gómez‐Urbano, Markus Binder, Endy Nugroho Dwiputra, Thomas Diemant, Dominic Bresser, Adriano Pierini, Andrea Cioffi, Enrico Bodo, Sergio Brutti, Matjaž Koželj, Tom Gouveia, Emma Bremond, Alix Ladam, Sébastien Fantini, Susan Sananes‐Israel, Imanol Landa‐Medrano, Iratxe de Meatza, Andrea Balducci

**Affiliations:** ^1^ Institute For Technical Chemistry and Environmental Chemistry and Center for Energy and Environmental Chemistry Friedrich‐Schiller University Jena Germany; ^2^ Helmholtz Institute Ulm (HIU) Electrochemical Energy Storage Ulm Germany; ^3^ Karlsruhe Institute of Technology (KIT) Karlsruhe Germany; ^4^ Ulm University (UUlm) Ulm Germany; ^5^ Department of Chemistry Sapienza University of Rome Rome Italy; ^6^ Consiglio Nazionale delle Ricerche Istituto Dei Sistemi Complessi Rome Italy; ^7^ Solvionic Toulouse France; ^8^ CIDETEC, Basque Research and Technology Alliance (BRTA) Donostia Spain

**Keywords:** electrolyte, cathode, lithium‐ion battery, NMC, sustainability

## Abstract

This study reports the formulation of innovative electrolytes designed to improve safety, sustainability, and compatibility with LiNi_0.92_Mn_0.04_Co_0.04_O_2_ (NMC92) cathodes. The use of bio‐based solvents (γ‐valerolactone, GVL) and safe, stable, innovative co‐solvents (diethylene glycol butyl ethyl ether, DEGBEE) is investigated in combination with imide‐ and borate‐based salts, demonstrating reduced flammability and enhanced transport properties. Molecular dynamics simulations reveal their advantageous solvation characteristics, highlighting increased lithium‐ion mobility in GVL‐based electrolytes due to diminished ionic clustering. Overall, the investigated electrolytes exhibit outstanding electrochemical performance with NMC92 cathodes in a half‐cell configuration, retaining above 80% of their initial capacity after 300 galvanostatic cycles at 1 C and an enhanced rate capability. This behavior is attributed to the inorganic nature of the resulting cathode‐electrolyte interphase, as confirmed by ex situ x‐ray photoelectron spectroscopy. Finally, the suitability of this novel formulation for real‐scale application is evaluated at the pouch‐cell level, demonstrating similar performance to benchmark formulations while enhancing overall device safety and sustainability.

## Graphical abstract

This study reports the formulation of innovative electrolytes to improve safety, sustainability, and compatibility with Ni‐rich cathodes through the use of a bio‐based solvent (γ‐valerolactone, GVL) and a safe, stable, innovative co‐solvent (diethylene glycol butyl ethyl ether, DEGBEE). A complete analysis moving from physicochemical characterization and modeling to electrochemical laboratory‐scale testing and upscaled pouch cells is presented.

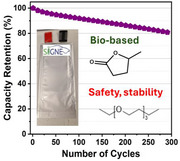

## 1 Introduction

Lithium‐ion batteries (LIBs) are critical for the electrification of energy and transportation systems, motivating significant efforts to increase their energy density. Among the various strategies, the development of high‐voltage cathode materials (e.g., lithium nickel manganese cobalt oxide, NMC; lithium nickel manganese oxide, LNMO) is particularly promising [1, 2, 3]. However, operating at higher voltages brings inherent safety concerns, including increased flammability and thermal runaway risks. From this perspective, thoughtful electrolyte design is essential to ensure safety and adequate interfacial performance [4, 5, 6]. Unfortunately, commercial formulations rely on the use of flammable solvents consisting of mixtures of carbonates such as ethylene carbonate (EC) and dimethyl carbonate (DMC) [7]. In addition, the benchmark lithium hexafluorophosphate (LiPF_6_) salt brings serious risks associated with its poor thermal stability and high fluorine content, potentially leading to the formation of hazardous species such as HF or POF_3_ [8]. These components pose additional environmental challenges, as their manufacture relies on resource‐intensive and hazardous processes [9]. Carbonate‐based solvents are industrially derived from fossil feedstocks or natural gas, while intensive mining (i.e., phosphate rock, lithium spodumene, fluorite) is required for the production of LiPF_6_ [10]. On this basis, further development of electrolytes from a safety and sustainability perspective is imperative to advance LIBs. In this regard, imide‐based salts lithium bis(trifluoromethanesulfonyl)imide (LiTFSI) and lithium bis(fluorosulfonyl)imide (LiFSI) present promising opportunities [11]. Besides their well‐known positive features for cell operation, these salts demonstrate enhanced thermal and hydrolytic stability when compared to LiPF_6_ [12, 13]. Among them, LiFSI has recently attracted attention since advances in its large‐scale production have led to a gradual increase in its portion in commercial electrolyte formulations [14]. Unfortunately, imide‐based salts are unable to protect the Al current collector from anodic dissolution at high potentials [15, 16]. This challenge can be solved through the incorporation of film‐forming additives, such as lithium difluoro(oxalato)borate (LiDFOB), to improve cell performance [17, 18]. On the other hand, solvent selection should be likewise guided following these sustainability and safety principles by prioritizing materials with enhanced flash points and bio‐derived origin. Previous studies have demonstrated the use of novel solvents such as 1,1,2,2‐tetraethoxyethane (TEG) with Ni‐rich cathodes [19]. The formulation of TEG‐based electrolytes offers several benefits for NMC half‐cell operation due to its favorable Li coordination, film‐forming ability, and reduced flammability [20]. Nevertheless, the acetal nature of TEG makes it susceptible to nucleophile attack, resulting in ethanol elimination and the formation of heavily colored products. This can compromise the long‐term operation and shelf life of formulations containing this chemical. In view of its good performance, novel solvents containing ether groups but exhibiting enhanced stability may offer promising performance for high‐voltage LIBs. In this regard, the barely explored diethylene glycol butyl ethyl ether (DEGBEE) represents a promising candidate due to its elevated flash point, chemical stability and coordination abilities, even if its application in LIBs remains almost unexplored in literature [21]. A common drawback of high‐flash‐point ether solvents (e.g., TEG, DEGBEE) is their elevated viscosity, which can hinder ionic transport. Blending them with low‐viscosity solvents could be an effective measure to counterbalance this challenge. In this regard, a potential alternative to conventional carbonates is γ‐valerolactone (GVL). While benchmark solvents hold the potential to be produced from renewable feedstocks, GVL is already bio‐derived from levulininc acid, one of the top 12 high‐value biomass‐derived platform chemicals [22, 23]. In addition to its renewable origin, GVL exhibits favorable solvent properties, such as a wide liquid range (−31°C to 207°C), a high flash point (96°C), low toxicity (LD_50_, oral, rat: 8800 mg kg^−1^), and good biodegradability [24]. Recently, its use has been successfully demonstrated in various energy storage devices [25, 26, 27, 28]. Moreover, the challenge of forming a stable solid electrolyte interphase (SEI) in GVL‐based electrolytes can be effectively addressed by the incorporation of suitable additives [29]. To the best of our knowledge, the use of GVL with Ni‐rich cathodes has not yet been reported.

Considering the above, this study proposes a strategic selection of new electrolyte components for use with Ni‐rich LiNi_0.92_Mn_0.04_Co_0.04_O_2_ (NMC92) cathodes, focusing on safety and sustainability while maintaining performance. In line with our previous findings [19], LiFSI and LiDFOB salts were fixed in the formulation, while fluoroethylene carbonate (FEC) was included to ensure suitable interfacial properties [30]. New solvent systems featuring DEGBEE and TEG as co‐solvents, together with GVL or propylene carbonate (PC) as the primary solvents were evaluated. This study reports a detailed analysis of their physicochemical and electrochemical properties, as well as their coordination behavior. Also, their compatibility with NMC92 half‐cells was assessed while providing insights on the structural and chemical evolution of the cycled electrodes through a comprehensive ex situ analysis featuring x‐ray photoelectron spectroscopy (XPS). Finally, the suitability of the most promising electrolyte formulation for large‐scale implementation was explored through the assembly of LIB pouch cells.

## 2 Results and Discussion

### 2.1 Physicochemical Characterization of the Electrolytes

The electrolytes were formulated by employing different solvent blends while keeping LiFSI as the main conducting salt and FEC/LiDFOB as additives. The studied solvent mixtures combined a main solvent (PC or GVL) and a co‐solvent (TEG or DEGBEE). The exact composition of the electrolytes formulated in this study is specified in Table 1, and the most relevant properties of their corresponding neat solvents are listed in Table 2.

**TABLE 1:** Electrolyte composition of the formulations employed in this study.

Electrolyte composition	Abbreviation
1 M LiFSI in **TEG/PC**/FEC (6:14:3, weight) + 2 wt.% LiDFOB	**TEG/PC**
1 M LiFSI in **TEG/GVL**/FEC (6:14:3, weight) + 2 wt.% LiDFOB	**TEG/GVL**
1 M LiFSI in **DEGBEE/PC**/FEC (6:14:3, weight) + 2 wt.% LiDFOB	**DEGBEE/PC**
1 M LiFSI in **DEGBEE/GVL**/FEC (6:14:3, weight) + 2 wt.% LiDFOB	**DEGBEE/GVL**
1 M LiPF_6_ in EC/DMC (1:1, volume) + 10 wt.% FEC + 2 wt.% VC + 1 wt.% LiTFSI	**Reference electrolyte**

**TABLE 2:** List of relevant parameters for the solvents employed in this study.

Solvent properties	PC [31, 32]	GVL [33, 34]	TEG [35, 36]	DEGBEE
Chemical structure	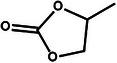	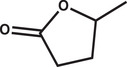	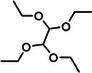	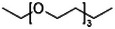
Chemical formula	C_4_H_6_O_3_	C_5_H_8_O_2_	C_10_H_22_O_4_	C_10_H_22_O_3_
Melting temperature [°C]	−49	−31	−35	−58
Boiling temperature [°C]	242	207	240	238
Flash point [°C]	132	96	71	86
Dielectric constant^a^	64.9	36.5	—	—
Viscosity [mPa s]^a^	2.5	2.0	1.74	1.61
Density [g cm^−3^]^a^	1.2	1.05 [33]	0.9	0.89
LD_50_, oral for rat [mg kg^−1^]	> 5000	8800	2000	—

^a^ Values at 25°C.

**FIGURE 1:**
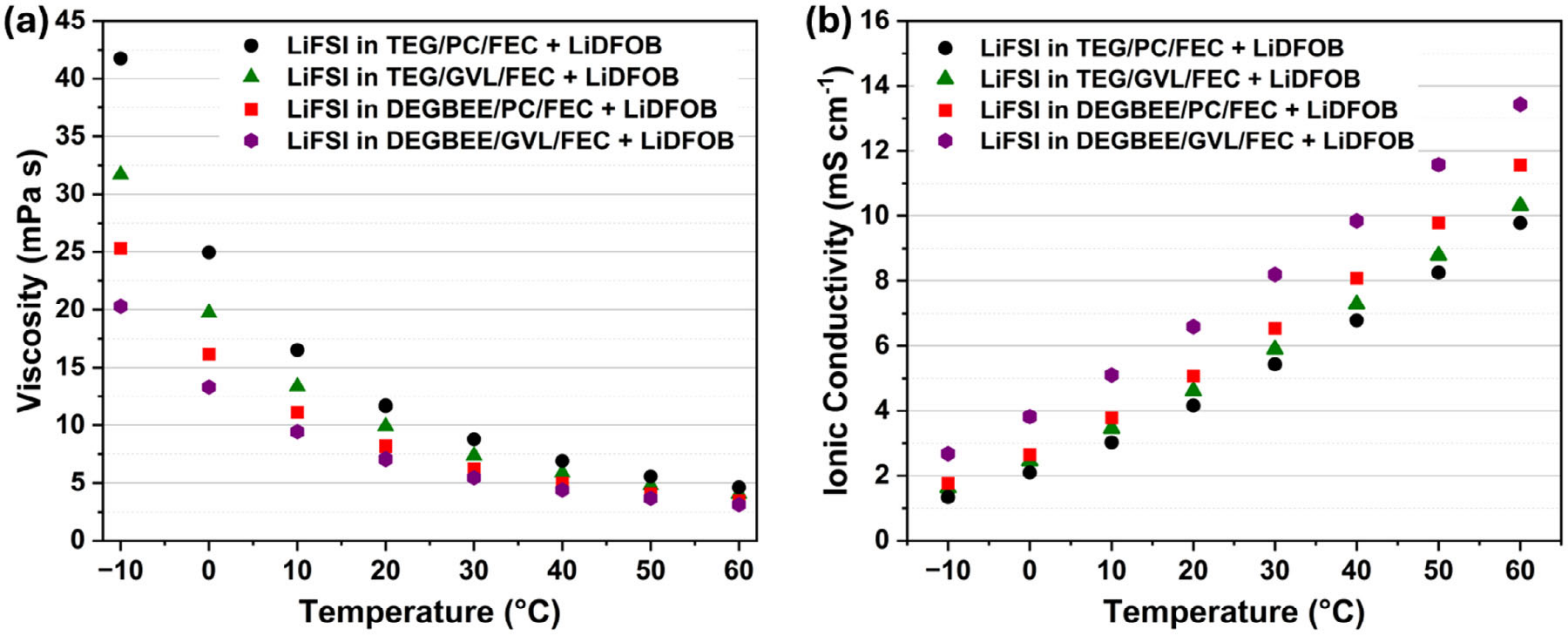
(a) Viscosity and (b) conductivity values measured as a function of temperature (−10°C to 60°C) for the noted electrolyte compositions.

The viscosity of the electrolytes was investigated across a temperature range from −10 to 60°C, as shown in Figure 1a. As expected, in all cases, their viscosity decreased with increasing temperature. The electrolytes containing DEGBEE as a co‐solvent displayed lower viscosity values compared to those with TEG. Also, the use of GVL over PC led to slightly lower viscosities. Accordingly, the lowest and highest viscosity values were measured for the DEGBEE/GVL and TEG/PC formulations, with 7.00 and 11.71 mPa s at 20°C, respectively (Table 3). The conductivity values of the electrolyte solutions (Figure 1b) followed the exact opposite trend to the viscosity for varying temperatures. The incorporation of DEGBEE and GVL demonstrated a positive impact on conductivity compared to TEG and PC. Specifically, the DEGBEE/GVL formulation achieved a conductivity of 6.58 mS cm^−1^, while its TEG/PC counterpart displayed 4.15 mS cm^−1^ at 20°C (Table 3). Overall, the trend in transport properties (increased conductivity, reduced viscosity) is: DEGBEE/GVL > DEGBEE/PC > TEG/GVL > TEG/PC. Despite representing a lower fraction of the total electrolyte composition, the co‐solvents (DEGBEE or TEG) had a higher impact than the main solvents (PC or GVL) on the measured transport properties.

**TABLE 3:** Overview of viscosity, conductivity, flash point, and density values at 20°C as well as the stability towards oxidation and reduction of the different electrolyte systems.

Electrolyte	Viscosity [mPa s]	Conductivity [mS cm^−1^]	Flash point [°C]	Density [g mL^−1^]	Ox. potential [V vs. Li^+^/Li]	Red. potential [V vs. Li^+^/Li]	Li^+^ transport number
TEG/PC	11.71	4.15	71	1.24	4.56	0.29	0.24 ± 0.04
TEG/GVL	9.92	4.61	75	1.17	4.85	0.18	0.32 ± 0.06
DEGBEE/PC	8.21	5.08	109	1.24	5.04	0.29	0.23 ± 0.07
DEGBEE/GVL	7.00	6.58	90	1.15	4.94	0.06	0.49 ± 0.02

In terms of safety, the measured flash points (*f*_p_) were also strongly influenced by the co‐solvent selection. While DEGBEE‐based formulations displayed a *f*_p_ of ca. 100°C, TEG‐based electrolytes remained around 70°C (Table 3). Overall, all the solutions showed an enhanced *f*_p_ relative to benchmark formulations like LP30 (*f*_p_: 31°C) [37], ensuring safer handling and storage. In addition, the investigated electrolytes displayed lower density values at 20°C than LP30 (1.29 g mL^−1^) [38], potentially reducing the electrolyte's weight contribution and thus enhancing the overall specific energy of the device. It is worth mentioning that GVL‐containing electrolytes showed slightly lower density values than their PC‐based counterparts (Table 3).

### 2.2 Electrolyte Modelling

**FIGURE 2:**
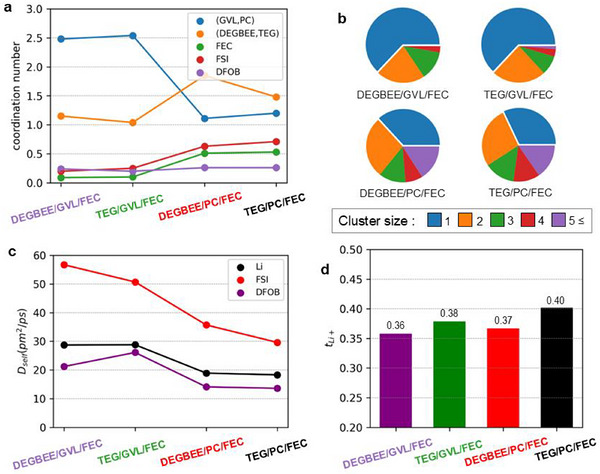
Analysis of electrolytes with FEC. (a) Coordination numbers of Li^+^ cations. (b) Analysis of ionic cluster sizes. Each pie‐plot reports the average fraction of total ions (Li^+^, FSI^−^, DFOB^−^) that participate in the formation of ionic clusters of different sizes: 1 = monomer (free ion), 2 = dimer (ion pair), 3 = trimer, 4 = tetramer, 5+ = pentamer and higher aggregates. (c) Self‐diffusion coefficients. (d) Lithium transference numbers.

To gain further insights into the dynamic properties of the electrolytes, molecular dynamics simulations were performed. The solvation and transport properties of the aforementioned electrolytes were examined using a polarizable force field, which has been previously validated for similar formulations (procedures in [20, 28]). In this work, the force field was integrated with additional parameters for FEC, DFOB^−^, and DEGBEE. The structural and dynamical analysis for the four studied formulations is summarized in Figure 2. Figure 2a reports the coordination environment of Li ions, showing clear differences in the extent and nature of ion–solvent and ion–anion interactions across the investigated formulations. The average Li^+^ coordination number of FEC molecules in the GVL‐based solvent is very low and increases for the PC formulations. The same holds for the anions, where in all formulations the Li^+^‐anion association is strongly disrupted, especially in the GVL‐based systems [28]. A partial association between Li^+^ and FSI^−^ survives in the PC‐based ones. The solvent‐Li^+^ and the Li^+^‐anion radial distribution functions across the studied electrolytes are shown in Figure S1. In Figure S1a in particular, the difference in the solvent average coordination of the Li^+^ can be noticed: the average number of GVL molecules in the first solvation shell is twice that of PC. This, in turn, allows more anions to enter the coordination sphere of Li^+^ (Figure S1b), where the different association degree with FSI^−^ is exemplified by the relative magnitude of the red and purple lines. No clear trends emerge in the solvating ability of the two cosolvents, with DEGBEE showing only slightly larger coordination numbers than TEG in the corresponding electrolytes. The dominant components of the Li^+^ solvation shell are the neutral molecules. GVL, when present, dominates over TEG or DEGBEE. On the contrary, in PC‐based solvents, the DEGBEE and TEG compete with PC in solvating the cation. In particular, the trends observed here suggest that variations of the neutral solvents significantly affect the degree of Li^+^ shielding, which is expected to play a central role in determining ion mobility and conductivity. Figure 2b complements this picture by quantifying the size of ionic clusters through a size‐resolved population analysis. An exemplary array of these ionic configurations (purged from the neutral molecules) is reported in Figure S2.

By monitoring the relative abundance of ion aggregation in monomers (free ions), dimers, trimers, tetramers, and larger aggregates (5+), the analysis shows how each electrolyte composition promotes or suppresses extended ionic association. The presence of a large population of monomers in GVL‐based compositions is expected owing to the previous analysis: in GVL, ionic clustering is suppressed due to tight solvation by neutral components. The absence of higher‐order clusters indicates diminished electrostatic correlations. This typically leads to enhanced ion transport, which is favored by a predominance of free ions or small clusters and, ultimately, an efficient ionic dissociation. PC is far less effective at preventing ionic association and solvating Li^+^. The other neutral components (DEGBEE and TEG) partially compensate it, but their low concentration limits their impact. The result is that the ionic monomers’ concentration decreases in DEGBEE/PC and TEG/PC.

These microscopic features have a direct impact on the macroscopic diffusion of the ions, as summarized in Figure 2c, where the self‐diffusion coefficients of the different species are compared. DEGBEE/GVL and TEG/GVL that exhibit higher fractions of free ions display markedly improved mobility. The most mobile ion is the FSI^−^ owing to its low coordination ability. The trend for Li^+^ and DFOB^−^ is essentially the same. When moving from DEGBEE/GVL to TEG/PC, the drop in FSI^−^ diffusivity is approximately half, while the corresponding decrease in Li^+^ mobility is limited to roughly one third. This correlates well with the calculated transport numbers ($t_{Li^{+}}$) reported in Figure 2d. The relatively small variation of the $t_{Li^{+}}$ despite pronounced changes in absolute diffusivities (Figure 2c–d) stems from compensating shifts in anion mobility and ionic correlations. GVL‐containing electrolytes increase Li^+^ solvation by neutral molecules and suppress higher‐order ionic aggregates (Figures 2a–b and S1), which enhances Li^+^ self‐diffusion. Concurrently, the diminished electrostatic correlation permits greater FSI^–^ mobility; because $t_{Li^{+}}$ reflects the relative contribution of Li^+^ mobility to the total ionic transport, simultaneous increases in anion mobility partially offset the Li^+^ gain and produce only modest changes in $t_{Li^{+}}$.

An analogous analysis conducted for the formulations without FEC (Figure S3) shows that the overall behavior remains largely unchanged. Composition and calculated densities for the formulations with and without FEC are reported in Tables S1 and S2, respectively.

### 2.3 Electrochemical Characterization of the Electrolytes

**FIGURE 3:**
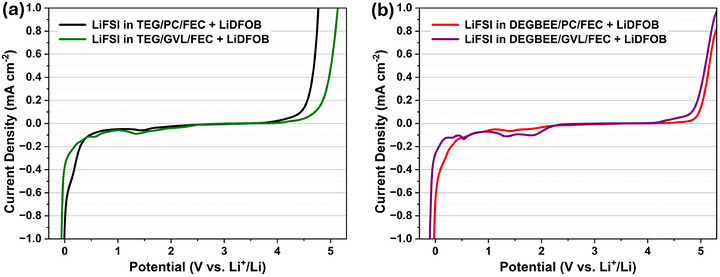
ESW determination of (a) TEG‐based and (b) DEGBEE‐based formulations.

The electrochemical stability window (ESW) of the electrolyte solutions was investigated through linear sweep voltammetry (LSV) to confirm their compatibility with nickel‐rich NMC92 electrodes. As shown in Figure 3a, the TEG/GVL formulation demonstrated greater stability against reduction (0.18 vs. Li^+^/Li) and oxidation (4.85 V vs. Li^+^/Li) than TEG/PC (0.29 to 4.56 V vs. Li^+^/Li). Conversely, DEGBEE‐based formulations exhibited greater stability against oxidation (Figure 3b). More specifically, DEGBEE/GVL displayed the widest ESW among all formulations, ranging from 0.06 to 4.94 V versus Li^+^/Li. Considering these results, all electrolytes appear sufficiently stable at high potentials to be employed with NMC electrodes. It is worth noting that some electrochemical processes are observed for all formulations at potentials between 0.8 and 1.8 V versus Li^+^/Li. These can be ascribed to the electrochemical reduction of LiDFOB and FEC components [39, 40].

As discussed in the introduction, LiFSI faces the challenge of being unable to prevent anodic dissolution on Al current collectors. To determine the onset potential for anodic dissolution processes in the presence of the electrolytes investigated here, a staircase potential‐step chronoamperometry test was carried out from OCV to positive potentials using uncoated aluminum discs (Figure S4). An exponential increase in current is observed for all formulations when the applied potential reaches 4.4 V versus Li^+^/Li. These values are lower than those measured through LSV with an inert Pt surface (cf. Figure 3). Thus, the exponential current increase recorded at 4.4 V versus Li^+^/Li can be ascribed to the initiation of parasitic reactions, usually attributed to the anodic dissolution of the Al current collector. To further investigate anodic dissolution, a more severe test was conducted by sequentially applying a constant potential of 4.3 V versus Li^+^/Li (the cutoff potential for NMC electrodes) to uncoated aluminum discs (Figure S5). Under these conditions, no significant current evolution was registered for any of the evaluated electrolytes. These results indicate that LiDFOB effectively mitigates Al anodic dissolution, enabling the use of such formulations with NMC cathodes [19].

Lithium‐ion transport numbers were determined using the Bruce–Vincent–Evans method (Table 3). A higher $t_{Li^{+}}$ is measured for the GVL‐containing formulations, agreeing well with the coordination features observed for such electrolytes in Section 2.2. Notably, the DEGBEE/GVL sample shows the largest $t_{Li^{+}}$ with a value of 0.49. It is worth noting that the $t_{Li^{+}}$ values derived from molecular dynamics (Figure 2d) and from Bruce–Vincent–Evans measurements (Table 3) agree in overall magnitude, although differences in trend are observed. These discrepancies arise from the fundamentally different nature of the two approaches: MD‐derived values reflect intrinsic bulk ion mobility under equilibrium conditions, whereas the Bruce–Vincent–Evans method determines an effective transport number under applied bias in a real cell, where interfacial processes, polarization, concentration gradients, and ion correlations influence the result. Such effects, particularly relevant in PC‐rich systems with stronger ionic association, account for the observed deviations (≈10%–60%). Accordingly, the two sets of values should be regarded as complementary rather than directly equivalent.

### 2.4 NMC Half‐Cell Characterization

**FIGURE 4:**
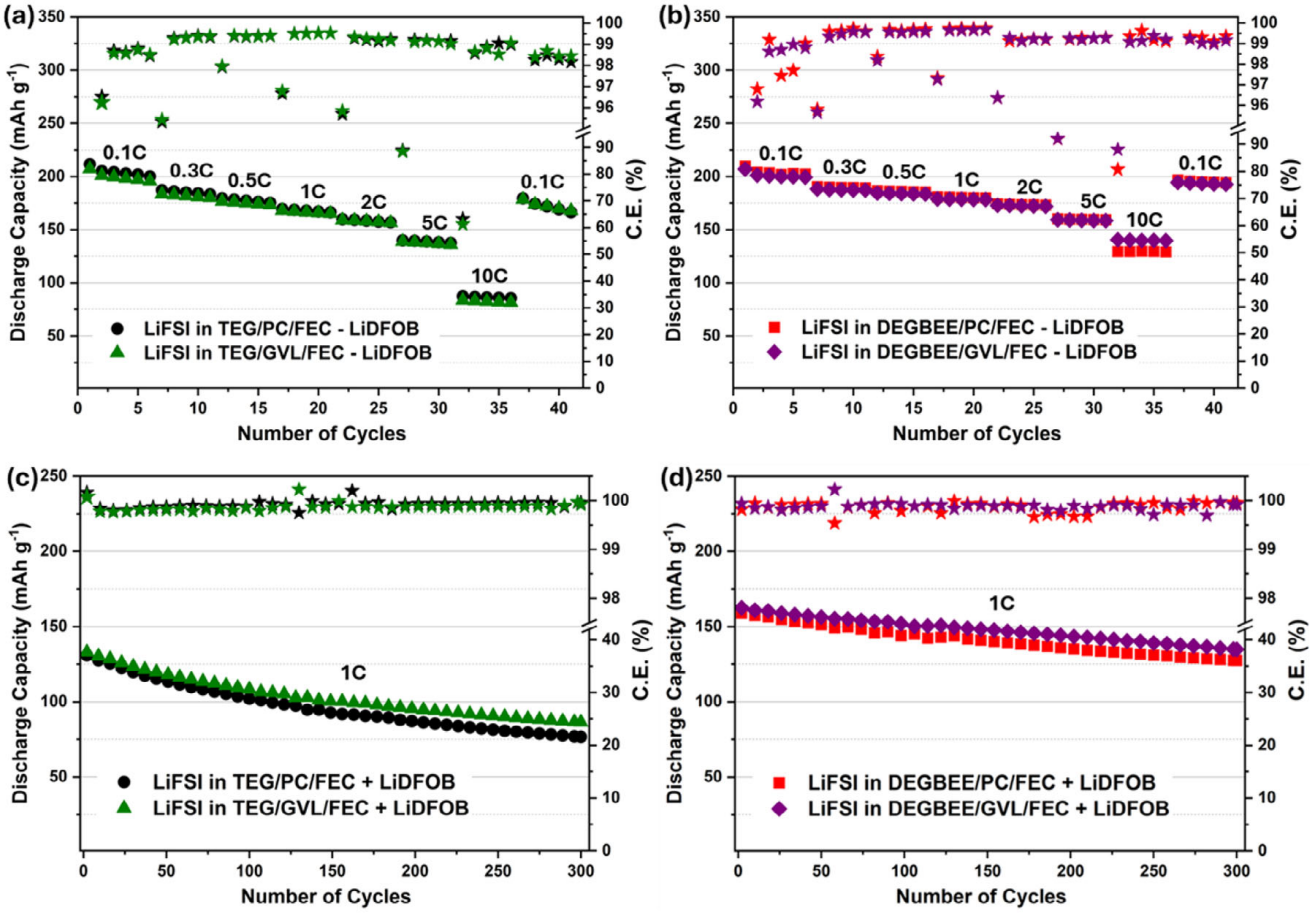
Rate capability tests performed for NMC92 electrodes in half‐cell configuration versus metallic lithium for (a) TEG‐ and (d) DEGBEE‐based electrolytes. Long‐term cycling at 1 C for corresponding (c) TEG‐ and (d) DEGBEE‐containing formulations.

After the physicochemical, computational, and electrochemical characterization of the electrolytes, their impact on the electrochemical performance of nickel rich NMC92 electrodes was investigated by galvanostatic cycling in half‐cell configuration. Details on cell components for the different electrochemical systems evaluated in this study are listed in Table S3. The voltage profiles of the first (at 0.05 C) and second (at 0.1 C) cycle are shown in Figure S6a–d. All curves display the characteristic signature profiles of Ni‐rich NMC cathodes [41]. The corresponding differential capacity curves (Figures S6e–h) reveal the characteristic peaks for the NMC delithiation at 3.8 V versus Li^+^/Li (hexagonal 1 to monoclinic), 4.0 V versus Li^+^/Li (monoclinic to hexagonal 2), and 4.2 V versus Li^+^/Li (hexagonal 2 to hexagonal 3). The opposite transitions were observed both at 0.05 and 0.1 C at 4.17 V versus Li^+^/Li, 3.97 V versus Li^+^/Li, and 3.75 V versus Li^+^/Li, respectively. First cycle coulombic efficiencies (CEs) of ca. 86% were obtained regardless of the formulation employed. In the subsequent cycle at 0.1 C, the CE increased to ca. 96% for all formulations, indicating good reversibility. Rate performance tests were subsequently conducted to TEG‐ (Figure 4a) and DEGBEE‐ (Figure 4b) containing electrolytes. Notably, all formulations delivered specific capacities of ca. 200 mAh g^−1^ during the initial cycles at 0.1 C. Nonetheless, an enhanced rate capability was observed for the DEGBEE‐based cells, particularly at higher C‐rates. In detail, the TEG/PC and TEG/GVL samples delivered ca. 140 mAh g^−1^ at 5 C, while DEGBEE/PC and DEGBEE/GVL reached ca. 160 mAh g^−1^. Slightly lower capacity and CE values were also observed for TEG‐containing electrolytes when restoring the C‐rate to 0.1 C (ca. 97.5%) compared to initial values (ca. 99%). This effect may be related to the acetal nature of TEG, making the electrolytes containing this molecule more susceptible to reacting and potentially compromising long‐term cycling performance. Regarding the influence of the main solvent, no significant differences in rate performance were observed when using PC instead of GVL. These findings align well with our previous works where we explored the use of such solvents in simplified electrolyte systems for LIBs, obtaining similar rate performances for PC‐ and GVL‐based formulations [26, 29]. Overall, the co‐solvent appeared to have a greater impact on the electrochemical performance than the primary solvent. This correlates well with the influence of the electrolyte components on the resulting transport properties, as shown in Figure 1. Nevertheless, it is worth mentioning that slightly better rate capabilities were achieved when combining the bio‐based GVL with the novel DEGBEE co‐solvent, reaching ca. 140 mAh g^−^^1^ at 10 C (Figure 4b). For the sake of comparison, the rate capability of a benchmark formulation (1 M LiPF_6_ in EC/DMC with VC, FEC and LiTFSI) was also evaluated (Figure S7a). This LiPF_6_‐based formulation demonstrated enhanced performance over the TEG‐based electrolytes at high current densities. Conversely, the formulations with DEGBEE overperformed this commercial electrolyte across the entire range of current densities. Following the C‐rate examination, the NMC92 half‐cells were subjected to galvanostatic cycling at 1 C for 300 cycles to evaluate their capacity retention. As already indicated by the rate capability tests, the potential occurrence of parasitic reactions related to the TEG component substantially reduced the measured capacity of the TEG‐containing electrolytes (Figure 4c). In contrast, the DEGBEE‐containing formulations exhibited not only higher capacity values but also enhanced cycle life (Figures 4d and S7b): DEGBEE/GVL retained 83% of its initial capacity after 300 cycles, followed by DEGBEE/PC (80%), TEG/GVL (65%) and TEG/PC (58%). In sum, these results show that DEGBEE and GVL improve the capacity and stability of the cathodes upon cycling. It is also worth noting that all formulations displayed stable CE values above 99.7% during this long‐term cycling test.

### 2.5 Ex Situ Characterization

To understand the compatibility of the new electrolyte formulations and the NMC92 cathodes in more detail, the cycled cathodes were ex situ characterized via scanning electron microscopy (SEM), energy dispersive x‐ray spectroscopy (EDX), and XPS analysis. Figure S8 shows the SEM pictures of the uncycled NMC92 cathode, while Figures S9–S13 show the SEM and EDX analysis of the cathodes after 300 cycles at 1 C in the aforementioned formulations. From the SEM images after cycling, we can clearly see the formation of surface species on the NMC particle surface. The deposits show very similar morphology regardless of the electrolyte employed (both for LiFSI‐ and LiPF_6_‐based formulations). This indicates that the CEI generated is comparable in thickness and density and that the new electrolyte formulations are generally compatible with such nickel‐rich cathode materials. The EDX results reveal a significantly higher fluorine concentration on the cathode for the LiPF_6_‐based electrolyte compared to the LiFSI‐based electrolytes. This observation is expected due to the higher fluorine content of PF_6_^−^ over FSI^−^. Noticeable differences in F content are also observed among the different LiFSI‐based electrolytes. Interestingly, the DEGBEE/PC and TEG/PC formulations show anomalous F content (0.52 and 25.1 wt.%, respectively) when compared to TEG/GVL and DEGBEE/GVL (6.1 and 6.0 wt.%).

**FIGURE 5:**
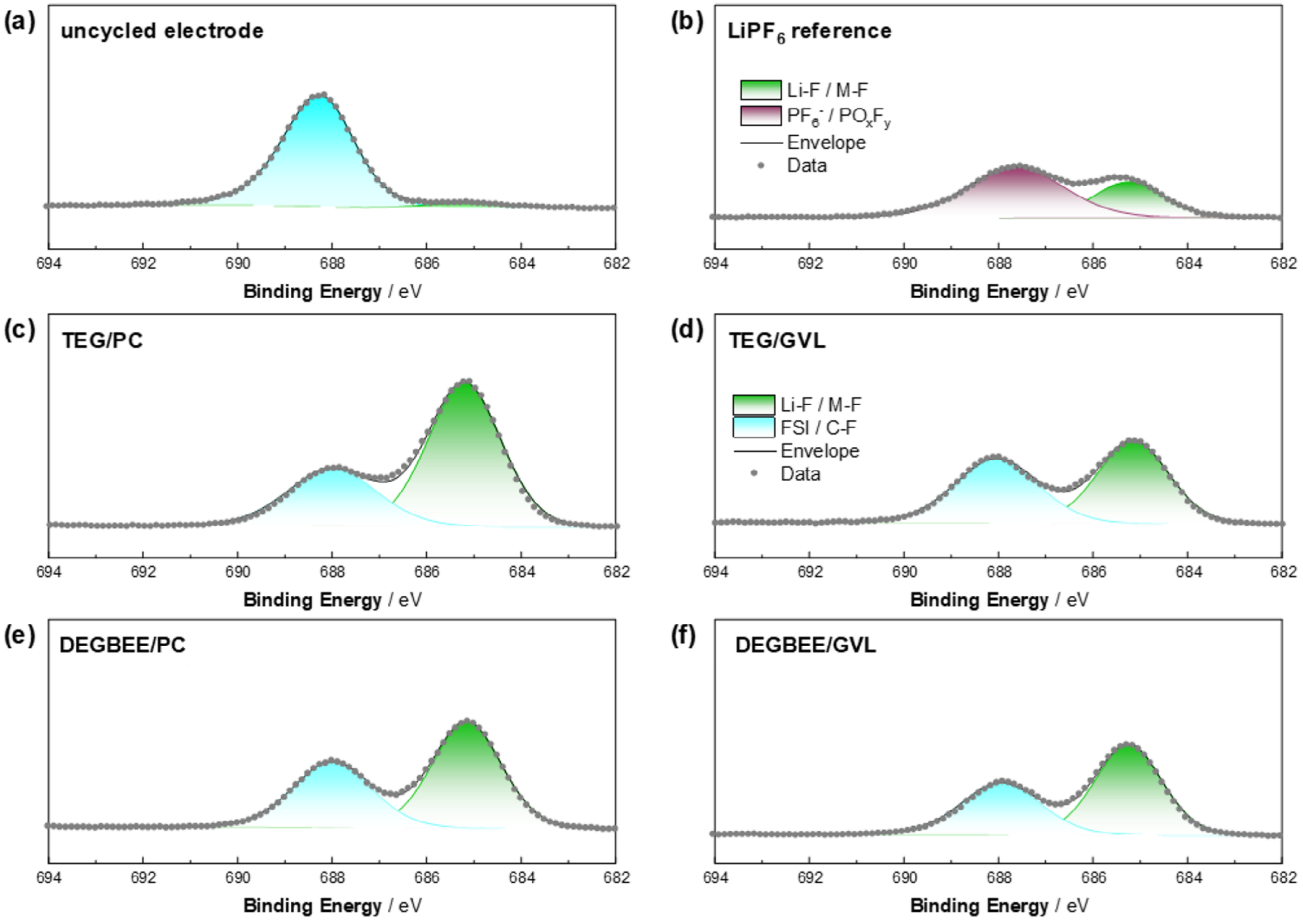
F1s region of the XPS detail spectra of the NMC92 cathodes recorded for (a) uncycled, or after 300 cycles at 1 C in (b) LiPF_6_ in DMC/EC, (c) LiFSI in TEG/PC, (d) LiFSI in TEG/GVL, (e) LiFSI in DEGBEE/PC, and (f) LiFSI in DEGBEE/GVL.

To further analyze the CEI, post‐cycling XPS measurements were performed (Figures 5 and S14–S17). Looking at the metal oxide peaks in the O 1s region (Figure S15), referring to the NMC lattice, we see a much more pronounced decrease in the intensity for the LiPF_6_‐based electrolyte, pointing to stronger coverage of the electrode material by the CEI. In contrast, the CEI layer in the LiFSI‐based electrolytes seems to be thinner, and hence a stronger metal oxide peak can be seen after cycling. The strong lattice coverage for the LiPF_6_‐containing formulation correlates with an intense C = O/O‐C = O signal, while the LiFSI‐based electrolytes show much less C = O/O‐C = O species on the cathode's surface. While all LiFSI‐based electrolytes show a lower content of oxygen‐containing organic CEI species in the surface layer (Table S4), a significantly higher amount of inorganic/metal fluorides like LiF is detected (Figure 5, Table S4). Such a thin inorganic CEI is believed to be beneficial for the cycling stability [42].

As noted from the NMC92 half‐cell measurements, the DEGBEE‐containing electrolytes enable higher capacity and more stable capacity retention upon cycling, compared to the electrolytes using TEG as co‐solvent. Comparing the XPS results of these two electrolyte groups, the most significant difference lies in the higher share of oxygen species for DEGBEE, indicating a stronger contribution of the solvents to the CEI.

In light of these results, one plausible explanation for the very low surface F content detected by EDX for DEGBEE/PC (versus the high F content for TEG/PC, Figures S10–S12 and Table S4) is that the co‑solvent alters oxidative decomposition pathways and product fate: TEG/PC may form poorly soluble, fluorine‑rich residues that accumulate at the immediate surface, whereas DEGBEE/PC may produce more oxygenated or more soluble/volatile fluorinated products, or a thinner/more organic outer CEI that masks subsurface fluorine species from the surface‑sensitive XPS probe.

### 2.6 LIB Pouch Cell

In view of the promising features of the 1 M LiFSI in DEGBEE/GVL/FEC with LiDFOB electrolyte (DEGBEE/GVL), its large‐scale implementation was explored in a LIB pouch cell configuration. First, its compatibility with graphite electrodes was assessed through galvanostatic cycling in half‐cell configuration versus metallic lithium at the lab‐scale. The conventional lithium intercalation and deintercalation staging mechanism in graphite was confirmed by the appearance of three distinct plateaus between 0.22 and 0.08 V versus Li^+^/Li (Figure S18a). In spite of the inability of GVL to form a stable SEI, no solvent co‐intercalation was observed due to the initial decomposition of LiDFOB and FEC during the first discharge cycle at ca. 1.6 and 0.8 V versus Li^+^/Li, respectively (Figure S18b). As a result of SEI formation, an initial CE of 88.1% was recorded in the first cycle at 0.05 C, which increased to 98.5% in the subsequent cycle at 0.1 C. The compatibility between this novel electrolyte and the graphite electrodes was further supported even at higher current density, delivering specific capacities above 350 mAh g^−1^ at 0.5 C (Figure S18c). The LiPF_6_‐based reference electrolyte was also measured in graphite half‐cells and exhibited similar initial coulombic efficiencies (89.3%) but slightly lower specific capacities (ca. 330 mAh g^−1^ at 0.5 C). However, it is worth noting that at 2 C, the capacity delivered by the reference electrolyte exceeded that of the DEGBEE/GVL formulation.

**FIGURE 6. (a):**
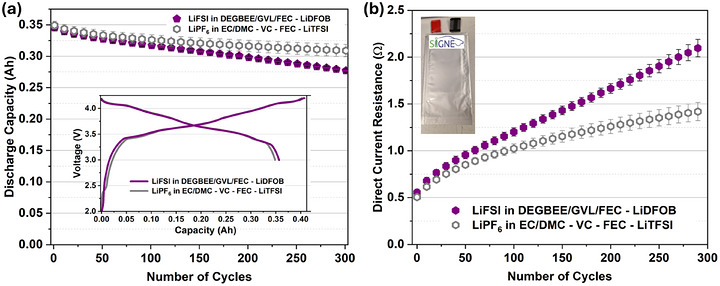
Galvanostatic cycling for noted electrolytes in a LIB pouch‐cell consisting of graphite negative electrodes and NMC811 positive electrodes, showing the capacity for the cycles at 0.1 C and the initial formation curves at 0.05 C in the inset. (b) Corresponding DCIR evolution for the cycles at 0.1 C with a picture of a pouch cell in the inset of the figure. Error bars correspond to the standard deviation obtained from three independent measurements.

For the LIB pouch‐cells assembly, DEGBEE/GVL electrolyte was employed in combination with a commercial NMC811 electrode as the positive electrode and a graphite‐based negative electrode. A pouch cell containing 1 M LiPF_6_ in EC:DMC 1:1 (vol/vol) + 10 wt.% FEC + 1 wt.% VC + 1 wt.% LiTFSI as the electrolyte was also assembled for the sake of comparison. The charge/discharge curves corresponding to the initial formation cycle at 0.05 C for both type of cells are depicted in the inset of Figure 6a. These are selected curves of one of the three cells tested per experiment. No significant differences were observed in the galvanostatic profiles obtained for the commercial electrolyte and the novel formulation, achieving acceptable CE values of 87.6 ± 0.9% and 86.9 ± 0.6%, respectively. Both devices delivered initial discharge capacities of 0.356 ± 0.006 and 0.352 ± 0.004 Ah, respectively. Notably, no significant gas formation was observed at this scale for both electrolytes, as shown in Figure S19. These results indicate that no side‐reaction between electrolyte and pouch cell materials is occurring, corroborating the good chemical stability, compatibility with the rest of the components, and scalability of DEGBEE/GVL electrolyte. Pouch cells were subsequently cycled at 0.3 C for 300 cycles, with one out of every 10 cycles performed at 0.1 C followed by a 1 C discharge pulse at 50% state‐of‐charge (SOC) to determine the direct current internal resistance (DCIR) of the cells. The promising features of DEGBEE/GVL are confirmed by the similar capacity evolution observed during the cycles performed at 0.1 C when compared to the commercial formulation (Figure 6a). Nevertheless, the LiPF_6_‐based electrolyte demonstrated better performance when considering the cycles conducted at 0.3 C (Figure S20). More specifically, while both DEGBEE/GVL and the commercial electrolyte retained more than 80% of the initial capacity after 300 cycles when considering the cycles performed at 0.1 C, this threshold was reached after around 250 cycles for the novel electrolyte when considering the capacity retention at 0.3 C. This difference can be mainly ascribed to the reduced transport properties (i.e., conductivity, viscosity) of DEGBEE/GVL when compared to those of commercial formulations employing carbonate‐based solvents (EC/DMC). This hypothesis matches well with the increase in the DC resistance (Figure 6b) of the cells with DEGBEE/GVL electrolyte, which was similar to the reference electrolyte at the beginning‐of‐life, but increased more significantly during the cycle life of the cells.

Interestingly, the results measured for the pouch cells do not correlate well with the lab‐scale NMC92 half‐cell observations in Section 2.4, where the DEGBEE‐based formulations outperformed the LiPF_6_‐based electrolyte in terms of capacity and capacity retention. The slightly inferior performance of the DEGBEE/GVL electrolyte in the full‐cell configuration was confirmed at lab scale using the same materials as in the half‐cell tests (NMC92//graphite), thereby excluding the possibility that this behavior originates from the use of commercial NMC811 in the pouch cell (Figure S21). These results highlight an important point: the difficulty on translating half‐cell results to full‐cell performance. Cross‐talk events that may not be critical in half‐cell configurations may play a decisive role in their full‐cell counterparts. Moreover, the loss of active lithium by LiPF_6_‐containing electrolytes could be a plausible explanation for its lower capacity retention (cf. Figure S7b) in NMC92 half‐cells [43, 44]. It is also worth noting relevant cell differences between half‐cells and LIB full‐cell pouch cells in terms of the separator employed, cell geometry or mass loadings (Table S3).

To further understand this mismatch, positive and negative electrodes were analyzed by XPS after the pouch cells reached 80% of their initial capacity (Figure S22, Table S5). Based on the signals of PVDF‐CF_2_ in the C 1s spectra and lattice oxide signal in the O 1s spectra, the thickness of the CEI at the cathode remains quite similar regardless of the electrolyte used. However, the SEI on the graphite anode is notably thinner after cycling with the DEGBEE/GVL electrolyte compared to the LiPF_6_‐based electrolyte. Most notably, the measurements revealed a lower fraction of LiF on the NMC811 cathode cycled in the DEGBEE/GVL electrolyte when compared to the cathode from the pouch‐cell cycled with the LiPF_6_‐based electrolyte. This contrasts with our previous half‐cell results, where the DEGBEE/GVL system showed a higher LiF content (Figure 5). Interestingly, XPS also revealed that the graphite anode cycled in the pouch‐cell with DEGBEE/GVL exhibited a significantly larger fraction of LiF, especially in comparison with the LiPF_6_‐based electrolyte. The higher LiF content at the pouch‐cell anode suggests that the DEGBEE/GVL electrolyte preferentially decomposes at the graphite surface, promoting SEI formation. This preferential decomposition at the anode may exhaust the film‐forming additives of the DEGBEE/GVL formulation, leading to gradual cathode‐side degradation due to the absence of a robust CEI. These results indicate that a careful optimization of the film‐forming additives is required to ensure an appropriate balance between SEI and CEI formation in the DEGBEE/GVL electrolyte. Regarding the O 1s spectra, slightly fewer ether (C–O)‐based decomposition products are observed at the cathode for the LiFSI electrolyte, along with a lower amount of carbonate (C = O)‐based decomposition products at the anode. This suggests improved stability of the LiFSI in the DEGBEE/GVL electrolyte in slowing down or reducing solvent decomposition. Overall, these findings highlight the stabilizing effect of the LiDFOB additive in promoting controlled CEI/SEI formation during full‐cell cycling. Overall, the results presented herein for DEGBEE/GVL in LIB pouch cells are very promising. This novel electrolyte almost matches the performance of conventional, well‐stablished formulations while offering several advantages from both safety and environmental perspectives as (i) it relies on the use of bio‐based GVL, (ii) avoids the use of LiPF_6_, aligning with ongoing legislation aimed to reduce the F content in commercial batteries, and (iii) its flash point is above 90°C, which ensures enhanced safety. Finally, this work explores the use of DEGBEE, which has demonstrated promising performance, especially for applications with nickel‐rich high‐capacity layered oxide‐based cathodes.

## 3 Conclusions

This work reports on the guided formulation of safe and sustainable electrolytes compatible with nickel‐rich NMC92 cathodes. Solvent mixtures incorporating an innovative co‐solvent (DEGBEE) and bio‐based alternatives (GVL) demonstrated adequate transport properties and enhanced safety due to their low flammability. Despite representing a lower fraction of the electrolyte composition, the investigated co‐solvents (DEGBEE or TEG) had a higher impact on the transport properties and flash points of the formulations than the primary solvents (PC or GVL). The combination of these solvents with a rational selection of salts (LiFSI, LiDFOB) and additives (FEC) led not only to enhanced ESWs compatible with NMC92 cathodes but also mitigated anodic dissolution processes and ensured good interfacial properties for both positive and negative electrodes. Among the different solvents evaluated, the best features in terms of transport properties (i.e., viscosity, conductivity), ESW, safety (i.e., flash point), and coordination abilities (modelling studies) were found for the formulation containing DEGBEE as the cosolvent and GVL as the primary solvent. As a result, this formulation demonstrated the best electrochemical performance in NMC92 half‐cells, delivering ca. 160 mAh g^−1^ at 5 C while retaining above 80% of its initial capacity after 300 charge/discharge cycles at 1 C. The ex situ characterization of the cycled electrodes provided insights into the film‐forming abilities of the selected electrolytes. All formulations displayed a thick inorganic CEI layer with a high content of LiF. Notably, the portion of oxygen‐containing species was more significant for the DEGBEE‐containing electrolytes, indicating that this solvent effectively contributes to the formation of the CEI. Additionally, the DEGBEE/GVL electrolyte demonstrated compatibility with graphite‐based electrodes, delivering up to 350 mAh g^−1^ at 0.3 C in a half‐cell configuration. Finally, the 1 M LiFSI in DEGBEE/GVL/FEC with LiDFOB electrolyte (DEGBEE/GVL) was explored at a larger scale in a LIB pouch cell configuration. Despite showing slightly inferior capacity retention at 0.3 C, our novel formulation was able to deliver similar performance to a conventional LiPF_6_‐based electrolyte. Overall, this novel electrolyte demonstrates that a strategic combination of selected components can simultaneously integrate safety, sustainability, and reduced fluorine content without a substantial decline in performance. In particular, it incorporates bioderived GVL, avoids the use of LiPF_6_, and exhibits improved electrochemical stability thanks to the incorporation of DEGBEE, an innovative ether solvent with a high flash point. Also, to the best of our knowledge, this work includes the first report of GVL with Ni‐rich cathodes.

## Conflicts of Interest

The authors declare no conflicts of interest.

## Supporting information



Supporting File: anie72288‐sup‐0001‐SuppMat.docx.

## Data Availability

The data that support the findings of this study are available from the corresponding author upon reasonable request.
