# Supplementary material for: Rational Formulation of Ether‐Lactone Electrolytes for Safe and Sustainable Ni‐Rich Lithium‐Ion Batteries
Source: Angew Chem Int Ed Engl. 2026 Apr 24;65(24):e26145. doi: 10.1002/anie.202526145 (PMC13245610; doi:10.1002/anie.202526145)
Supplement: Supplementary file 1 — Supporting File: anie72288‐sup‐0001‐SuppMat.docx. [file ANIE-65-e26145-s001.docx]

**Supporting Information**

**Rational Formulation of Ether-Lactone Electrolytes for Safe and Sustainable Ni-Rich Lithium-Ion Batteries**

Juan Luis Gómez Urbano*^a^, Markus Binder^b,c^, Endy Nugroho Dwiputra^b,c^, Thomas Diemant^b,c^, Dominic Bresser^b,c,d^, Adriano Pierini^e^, Andrea Cioffi^e^, Enrico Bodo^e^, Sergio Brutti^e,f^, Matjaž Koželj^g^, Emma Bremond^g^, Alix Ladam^g^, Sebastien Fantini^g^, Susan Sananes-Israel^h^, Imanol Landa-Medrano^h^, Iratxe de Meatza^h^, Andrea Balducci*^a^

^a^ Institute for Technical Chemistry and Environmental Chemistry and Center for Energy and Environmental Chemistry, Friedrich-Schiller University Jena, Philosophenweg 7a, 07743 Jena, Germany

^b^ Helmholtz Institute Ulm (HIU) Electrochemical Energy Storage, Helmholtzstrasse 11, 89081 Ulm, Germany

^c^ Karlsruhe Institute of Technology (KIT), P.O. Box 3640, 76021 Karlsruhe, Germany

^d^ Ulm University (UUlm), 89069 Ulm, Germany

^e^ Department of Chemistry, Sapienza University of Rome, Piazzale Aldo Moro 5, 00185 Rome, Italy

^f^ Consiglio Nazionale delle Ricerche, Istituto dei Sistemi Complessi, Piazzale Aldo Moro 5, 00185 Rome, Italy

^g^ Solvionic, 11 Chemin des Silos, 31100 Toulouse, France.

^h^ CIDETEC, Basque Research and Technology Alliance (BRTA), Po. Miramón 196, 20014 Donostia, Spain

E-mail: andrea.balducci@uni-jena.de and juanlu.gomez.urbano@uni-jena.de

**Keywords:** Electrolyte; Cathode; NMC; Lithium-ion Battery; Sustainability

**Experimental Section**

*E.1 Chemicals and materials*

The solvent 1,1,2,2-tetraethoxyethane (**TEG**, CAS: 3975-14-2, WeylChem) was passed before use through a freshly activated alumina column (Brockmann I) in order to remove the stabilizer. It was subsequently dried with freshly activated molecular sieves (4 Å) until water content was reduced below 1 ppm. Diethylene glycol butyl ethyl ether (**DEGBEE**, CAS: 3895-17-8) was synthetized through a Williamson ether coupling from di(ethylene glycol) ethyl ether and 1-bromobutan. After distillation, a colorless liquid was collected with 97% yield. DEGBEE was dried with freshly activated molecular sieves (3 Å) until water content was reduced below 1 ppm. Nuclear magnetic resonance (NMR) of DEGBEE was analyzed using a Magritek Spinsolve 80 MHz. Product structure was found in agreement with: ^1^H NMR (80 MHz, neat): δ 0.66-0.91 (3H, triplet, CH_3_), 0.91-1.08 (3H, triplet, CH_3_), 1.14-1.69 (4H, multiplet, 2CH_2_), 3.15-3.51 (12H, multiplet, 6CH_2_). ^13^C NMR (80 MHz, neat): δ 13.40, 14.80, 19.09, 31.80, 65.91, 69.80, 70.08, 70.48. Electrolytes were prepared by mixing 1 M lithium bis(fluorosulfonyl)imide (**LiFSI**) in DEGBEE/TEG, propylene carbonate (**PC**)/gamma-valerolactone (**GVL**), and fluoroethylene carbonate (**FEC**), with respectively 6:14:3 wt.% and 2 wt.% of lithium difluoro(oxalato)borate (**LiDFOB**). LiFSI (CAS: 171611-11-3, 99.9%, <20ppm H_2_O), lithium bis(trifluoromethanesulfonyl)imide (**LiTFSI**, CAS: 90076-65-6, 99.9%, <20ppm H_2_O), LiDFOB (CAS: 409071-16-5, 99.9%, <20ppm H_2_O), vinylene carbonate (**VC**, CAS: 872-36-6, 99.99%, <20ppm H_2_O) and FEC (CAS: 409071-16-5, 99.9%, <20ppm H_2_O) were provided by Solvionic. A solution of 1 M LiPF_6_ in EC:DMC (1:1 vol.%) with 10 wt.% FEC, 2wt.% VC and 1 wt.% LiTFSI provided by Solvionic was also used as a reference electrolyte. Metallic lithium employed in this study was purchased from China Energy Lithium Co., Ltd.

*E.2 Physicochemical characterization*

Viscosity measurements were carried out with a MCR 102 Rheometer equipped with a CP50-0.5 cone-plate system from Anton Paar in a temperature range of -20 °C to 60 °C with a constant shear rate of 1000 s^-1^. The sample (0.5 mL) application was done at 20 °C. The conductivity of the electrolytes was measured with a Modulab XM ECS potentiostat in combination with a Binder MK53 climate chamber. Alternating current resistance was determined within a temperature range from -30 to 80 °C in a conductivity cell consisting of two parallel platinum electrodes. The determination was done at open circuit voltage by applying an alternating voltage with an amplitude of 5 mV, within a frequency range from 300 kHz to 1 Hz. The electrolyte volume for each measurement was 0.5 mL. Conductance was calculated through the reciprocal of the alternating current resistance. The conductivity values were obtained after multiplying the as-obtained conductance by the known-cell constant. The cell constant was determined by employing a standardized 3 M KCl solution. Lithium ion transport numbers were obtained by using the so-called Bruce-Vincent-Evans method where a chronoamperometric test (voltage bias 20 mV, test time 60 minutes) is carried out between two EIS scans (frequency range 100kHz-1mHz, voltage bias 10 mV) by using an IVIUM-Vertex instrument and a symmetric Li/Li cell (ECC-STD El-cell) with a Whatman separator soaked by 0.05 mL of electrolyte. Tests were performed at least four times on different cell assemblies. Densities of the electrolytes were determined by a falling ball viscosimeter Lovis 2000. Flash points were measured by an Eraflash automated flash point tester, using the continuously closed cup flash point testing method according to ASTM D7094.

*E.3 Lab-scale electrodes and cell preparation*

For lab-scale testing, NMC92 (LiNi_0.92_Mn_0.04_Co_0.04_O_2_) cathode active material was employed. This material was synthesized using a hydroxide precursor, lithium hydroxide (LiOH) as the lithium source, and a two-step calcination. First, a molten salt reaction at 850 °C (12 h) in pure oxygen atmosphere employing Li_2_SO_4_ as flux agent was performed (transition metal : Li ratio 1:2.5). After washing off the excess salts with demineralized water, a second calcination at 700 °C (12 h) was performed to obtain the final NMC92. The electrodes were prepared using 92 wt.% NMC92, 4 wt.% conductive carbon (CNERGY Super C65, Imerys) and 4 wt.% polyvinylidene difluoride (**PVdF**, Solef 6020, Solvay) binder. The slurry was mixed with a planetary mixer (ARE-250, Thinky) and cast with a wet film thickness of 90 µm on battery-grade aluminum foil inside a dry room, dried at 120 °C for 12 h, and eventually calendered to a target porosity of 35%.

The graphite electrodes consisted of 94 wt.% natural graphite (Gr, GHDR15-4, Imerys Carbon & Graphite), 2 wt.% carbon black (C-NERGY Super C45, IMERYS Carbon & Graphite), 2 wt.% of carboxymethylcellulose (**CMC**, Walocel 2000, DOW Chemical Company, dissolved to 3 wt.%) and 2 wt.% styrene-butadiene-rubber (**SBR**, Al-3001 48%wt., Nippon A&L Inc.). These materials were mixed in deionized water using a mechanical mixer (RW 20 digital, IKA). Slurries consisted in 70 g solids and 60% solid content. Slurry preparation was performed at laboratory atmosphere at CIDETEC Energy Storage (22 °C, 50% relative humidity) and it was coated onto a 8 µm-thick copper foil (using doctor Blade). Wet electrodes were dried in a convection oven at 60 °C for 30 minutes before storage in dry conditions. The coatings were calendered to 1.45 g cm^−3^ using a table-top calendering machine (DPM solutions). The density of the coatings was determined by measuring their thickness using a micrometer (389–271C, Mitutoyo). Circular-shaped electrodes of 12 mm diameter were punched out of graphite- and NMC92-based castings. The resulting electrodes displayed an active material mass loading of 5.4 mg cm^-2^ for NMC92 and of 4.7 mg cm^-2^ for graphite. The electrochemical measurements were conducted using Swagelok-type cells. Circular Whatman GF/D glass microfiber filter sheets (CAT-No. 1823-150, 13 mm diameter) soaked with an electrolyte volume of 150 µL acted as separator between the working and counter electrode. In the case of the three-electrode setup an additional glass microfiber filter (8 mm diameter) was utilized as separator between the working/counter and reference electrodes. Cells were assembled in an argon-filled MBraun glovebox Lab Master Pro Eco (H_2_O < 0.1 ppm, 0_2_ < 0.1 ppm).

*E.4 Pouch-cell electrodes and cell preparation*

The electrodes used for pouch cell assembly were prepared in a semi-automatic roll-to-roll pilot coating line at CIDETEC facilities. The positive electrode formulation consisted of 95% LiNi_0.8_Mn_0.1_Co_0.1_O_2_ (NMC811, T81RC, BASF SE) as the active material, 2% carbon black (C-NERGY Super C65, IMERYS Carbon & Graphite) as the conductive additive, and 3% PVDF (Solef^®^ 5130, Solvay) as the binder. A slurry was prepared from 0.75 kg solid material using N-methyl pyrrolidone (NMP, synthesis grade, Scharlab, Spain) as the solvent and mixed in an EL 1 (Eirich) mixer. The slurry was coated on one side of the aluminium current collector (Hydro, 15 µm-thick) using a roll-to-roll coater with knife system (SC 30, COATEMA Coating Machinery GmbH). The coating was dried in the same line in three consecutive convection ovens at 110 °C, 120 °C, and 110 °C, respectively. The coating was calendered using a semi-industrial calendering machine (Naknor). The areal weight of the NMC811 electrode was 16.3 mg cm^-2^ (2.94 mAh cm^-2^), corresponding to a density of 3.2 g cm^-3^ (389–271C, Mitutoyo micrometer).

The graphite electrode formulation was also upscaled using the same instruments and materials, except for CMC (T2000GA, IFF/DuPont), SBR (BM-451B, Zeon) and the slurry mixer. A slurry with 600 g solids was prepared in a Speedmixer® (Hauschild) and coated on both sides of the copper current collector. The coating was dried in the same line in three consecutive convection ovens at 70 °C, 80 °C, and 70 °C, respectively. After calendering, the graphite electrode areal weight was 9.88 mg cm^-2^ per side (3.25 mAh cm^-2^) with 1.48 g cm^-3^ density. The total negative/positive ratio for electrode balancing was N/P=1.10 (10% anode capacity excess).

Electrodes manufactured in the semi-industrial coating lines were used to assemble pouch cells. Electrodes were obtained from these coatings using a semiautomatic die-cutting unit (MTI Corp., Richmond, VA, USA). The dimensions of the anodes and the cathodes were 10 cm × 6.1 cm and 9.8 cm × 5.9 cm, respectively. The electrodes were dried under vacuum at 120 °C while the aluminum laminated foil (ALF), the separator (Celgard H2010+CSP, laminable) and the flanges (tabs) were dried at 60 °C in a vacuum for 16 h. All these drying steps were conducted inside a dry room with a dew point of -40 °C. The electrodes were stacked manually inside this dry room using a guiding tool to guarantee the stack alignment. Each cell consisted of one double-side coated anode and two single-side coated cathodes. After stacking, the tabs of the electrodes were ultrasonically welded to terminal tabs (100 µm-thick Al (+) and Ni-plated Cu (−)). The stack was later placed in between two squared pieces of ALF. Three sides of these pieces of ALF were thermally sealed, while the other was used to add 2 mL of electrolyte, either with carbonate-based solvents (1 M LiPF6 in EC:DMC 1:1 + 10% FEC + 1% VC + 1% LiTFSI) or with DEGBEE/GVL. Each electrolyte was tested in three cells. After electrolyte filling, the cells were sealed under vacuum. Custom-designed cell holders, tailored to the specific pouch cell dimensions, were used for both formation and testing to ensure sufficient and uniform pressure distribution across all cell components (pressure applied: 2 kg cm^-2^). After formation, the cells were brought back to the dry room for the degassing step, after which they were sealed in vacuum again. Further information on pouch cell blueprints can be found in ^[1,2]^.

*E.5 Electrochemical measurements*

The electrochemical stability window of the electrolytes was analyzed using linear sweep voltammetry (LSV) at 5 mV s^‑1^ (± 0.2 mA cm^‑2^ current threshold). For this purpose, a Pt electrode was used as working electrode, a silver wire as quasi-reference electrode and a graphite rod as counter electrode. Separate LSV tests were conducted to determine the corresponding anodic and cathodic stability limits and ferrocene was used as internal standard for accurate potential determination. In addition, sequences of chronoamperometric measurements were carried out to investigate the onset potential of the electrochemical reactions (anodic dissolution) at an aluminum working electrode. For this purpose, the potential was gradually increased by 0.1 V increments from 3.5 V vs. Li^+^/Li to 5 V vs. Li^+^/Li, with a holding time of one hour for each potential step and recording the current response. These tests were performed in coin type cells, using lithium metal as anode (reference and counter electrode), a glass fiber separator soaked with the corresponding electrolyte, and a disc of aluminum as cathode (working electrode). Anodic dissolution tests were also performed in a three-electrode Swagelok cells employing an aluminum disk as working electrode and lithium metal discs as counter and reference electrodes. The potential of the aluminum discs was scanned at 0.5 mV s^‑1^ between 3.0 – 4.3 V vs. Li^+^/Li and held for 3 h at the upper potential limit in each cycle. A total of fifteen cycles were performed while the resulting current was recorded. NMC92 as well as graphite electrodes were evaluated in a half-cell configuration using a two-electrode Swagelok cell set up. In these measurements, either a NMC92 or a graphite electrode was utilized as working electrode and an oversized lithium metal electrode acted simultaneously as counter and reference electrode. Galvanostatic charge/discharge measurements were conducted at different C-rates, whereas 1 C corresponds to 200 mA g^-1^ for NMC92 and to 350 mA g^-1^ for graphite. In the case of the NMC92 half-cells, an asymmetric charge/discharge protocol (between 3.0 to 4.3 V vs. Li^+^/Li) was followed for the C-rate tests, fixing the charge rate to 0.33 C and varying the discharge rate from 0.33 C to 0.5 C, 1 C, 2 C, 5 C and 10 C. Long-term cycling was performed after the rate capability tests using a symmetric charge/discharge rate of 1 C for 300 cycles. For the graphite half-cells, a symmetrical charge/discharge protocol (between 0.005 to 2.0 V vs. Li^+^/Li) was applied.

Lab-scale LIBs (NMC92//graphite, N/P = 1.6) were cycled between 3.0 and 4.2 V with 1 C corresponding to 200 mA g⁻¹. A constant current–constant voltage (CCCV) protocol was applied. Specifically, charging was performed under CC conditions up to 4.2 V, followed by a CV step until the current decreased to 50% of the applied CC value. Discharging was carried out under CC conditions down to 3.0 V. Specific capacities were calculated based on the total mass of the cathode active material. The LIB pouch cell characterization was started with a formation cycle at 0.05 C consisting of a CC charge until 4.2 V followed by CV until I<0.025 C. After degassing, a cycling protocol with check-up test was applied to these cells. This cycling protocol consisted of a repetition of the following sequence: 0.1 C symmetric cycle (CCCV charge, CC-discharge), 0.33 C to evaluate cell capacity, a 0.33 C cycle with a 1 C discharge pulse at 50% SOC, followed by 7 cycles at 0.33 C. The half-cell measurements were performed using a BioLogic VMP-3 multichannel potentiostat. The LIB pouch cells were tested using a Basytec Cell Test System potentiostat in a temperature-controlled room with 25 °C ± 1 °C controlled by air conditioning. The specific capacity and current density values were calculated with respect to the total mass of the cathode active material.

*E.6 Ex-situ characterization*

After cycling, the cells were opened in an argon-filled MBraun glovebox Lab Master Pro Eco (H_2_O < 0.1 ppm, 0_2_ < 0.1 ppm). The electrodes were cleaned by soaking with PC or GVL for approximately one minute and subsequently dried under vacuum at 40 °C overnight in a glass oven B-585 from Büchi. SEM analysis was conducted using a Zeiss Crossbeam XB340 field-emission electron microscope equipped with an energy-dispersive X-ray (EDX) spectroscopy detector (Oxford Instruments X-Max Xtreme, 100 mm^2^, 1−5 kV). For the SEM and EDX analysis, the acceleration voltage was set to 3 and 10 keV, respectively. Ex-situ X-ray photoelectron spectroscopy (XPS) measurements of the cycled NMC92 electrodes from lab-scale measurements and NMC811 cathodes and graphite anodes from the pouch cell tests were performed on a Specs XPS system with a Phoibos 150 energy analyzer. The spectra were acquired using monochromatized Al Kα radiation (300 W, 15 kV) and pass energies of 90 eV and 30 eV for the survey and the detail measurements, respectively. When necessary, sample charging was neutralized with an electron flood gun and the spectra were calibrated to the main C 1s peak at 284.6 eV of C=C/C-C species. To avoid surface contamination, the samples were transferred in an inert gas atmosphere from the glove box to the sample load lock of the XPS system. The peak fit of the XPS data was done with CasaXPS, using Shirley-type backgrounds and Gaussian-Lorentzian peak shapes.

*E.7 Molecular dynamics simulations*

Eight systems were simulated: DEGBEE/GVL (3:7 wt:wt), TEG/GVL (3:7 wt:wt), DEGBEE/PC (3:7 wt:wt) and TEG/PC (3:7 wt:wt) with LiFSI (1 m with respect to the main solvents mixture) and 2% LiDFOB with and without 10% FEC. The actual molality of Li^+^ is 1.05 for TEG mixtures and 1.07 for DEGBEE. The corresponding molality (owing to the LiDFOB salt addition decreases to 0.9-0.92. Classical molecular dynamics simulations have been performed using the polarizable AMOEBA model.^[3,4]^ The cell compositions and densities are reported in **Table S1** and **Table S2**. Initial random configurations were generated with the Packmol code.^[5]^ For each cell, the equilibration process was composed of a cell relaxation step at fixed NPT conditions (1 bar, 293 K) for 2 ns, followed by a thermalization step at fixed NVT (293 K) for 5 ns. The statistical sampling was run at fixed NVT for 5 ns, collecting frames every 0.05 ps. The Berendsen barostat and Bussi thermostat^[6,7]^ were employed to enforce constant pressure and temperature, with coupling constants of 2.0 ps and 0.1 ps, respectively. The two-timestep r-RESPA propagator^[8]^ was used to discretely propagate the classical equations of motion, applying a short dt = 0.25 fs for bonded interactions and a long dt = 2.0 fs for non-bonded interactions (for the NPT relaxation, the long timestep was 1.0 fs). The cutoffs for Ewald summation and vdW interactions were set to 7 Å and 12 Å, respectively.

The AMOEBA forcefield for PC, GVL, TEG, and FSI was already parametrized and validated in ^[9,10]^. The missing parameters for FEC, DEGBEE, and DFOB were obtained following the same procedure reported in the aforementioned papers.

Coordination numbers were calculated reading the running integral value of the radial distribution functions (RDFs) at a cutoff value of 2.75 Å, which results to be the first minimum in the overall RDF around Li cations. Analysis of ionic clusters was based on the method implemented by Frömbgen et al.^[11]^. Self-diffusion coefficients were estimated by linear fitting of the mean square displacement calculated for the Li atoms and the anions’ centers of mass.

*References experimental section*

[1] I. Landa-Medrano, A. Eguia-Barrio, S. Sananes-Israel, W. Porcher, K. Trad, A. Moretti, D. V. Carvalho, S. Passerini, I. De Meatza, “Insights into the Electrochemical Performance of 1.8 Ah Pouch and 18650 Cylindrical NMC:LFP|Si:C Blend Li-ion Cells” *Batteries* **2022**, *8*, 97.

[2] L. Lizaso, I. Urdampilleta, M. Bengoechea, I. Boyano, H.-J. Grande, I. Landa-Medrano, A. Eguia-Barrio, I. De Meatza, “Waterborne LiNi0.5Mn1.5O4 Cathode Formulation Optimization through Design of Experiments and Upscaling to 1 Ah Li-Ion Pouch Cells” *Energies* **2023**, *16*, 7327.

[3] J. W. Ponder, C. Wu, P. Ren, V. S. Pande, J. D. Chodera, M. J. Schnieders, I. Haque, D. L. Mobley, D. S. Lambrecht, R. A. DiStasio, M. Head-Gordon, G. N. I. Clark, M. E. Johnson, T. Head-Gordon, “Current Status of the AMOEBA Polarizable Force Field” *J. Phys. Chem. B* **2010**, *114*, 2549–2564.

[4] P. Ren, C. Wu, J. W. Ponder, “Polarizable Atomic Multipole-Based Molecular Mechanics for Organic Molecules” *J. Chem. Theory Comput.* **2011**, *7*, 3143–3161.

[5] L. Martínez, R. Andrade, E. G. Birgin, J. M. Martínez, “P ACKMOL : A package for building initial configurations for molecular dynamics simulations” *J. Comput. Chem.* **2009**, *30*, 2157–2164.

[6] H. J. C. Berendsen, J. P. M. Postma, W. F. Van Gunsteren, A. DiNola, J. R. Haak, “Molecular dynamics with coupling to an external bath” *J. Chem. Phys.* **1984**, *81*, 3684–3690.

[7] G. Bussi, D. Donadio, M. Parrinello, “Canonical sampling through velocity rescaling” *J. Chem. Phys.* **2007**, *126*, 014101.

[8] M. Tuckerman, B. J. Berne, G. J. Martyna, “Reversible multiple time scale molecular dynamics” *J. Chem. Phys.* **1992**, *97*, 1990–2001.

[9] A. Pierini, V. Migliorati, J. L. Gómez-Urbano, A. Balducci, S. Brutti, E. Bodo, “Simulations of γ-Valerolactone Solvents and Electrolytes for Lithium Batteries Using Polarizable Molecular Dynamics” *Molecules* **2025**, *30*, 230.

[10] A. Pierini, V. Piacentini, J. L. Gómez‐Urbano, A. Balducci, S. Brutti, E. Bodo, “A Polarizable Forcefields for Glyoxal Acetals as Electrolyte Components for Lithium‐Ion Batteries” *ChemistryOpen* **2024**, *13*, e202400134.

[11] T. Frömbgen, J. Blasius, V. Alizadeh, A. Chaumont, M. Brehm, B. Kirchner, “Cluster Analysis in Liquids: A Novel Tool in TRAVIS” *J. Chem. Inf. Model.* **2022**, *62*, 5634–5644.

**Section 1.** *Electrolyte modelling*


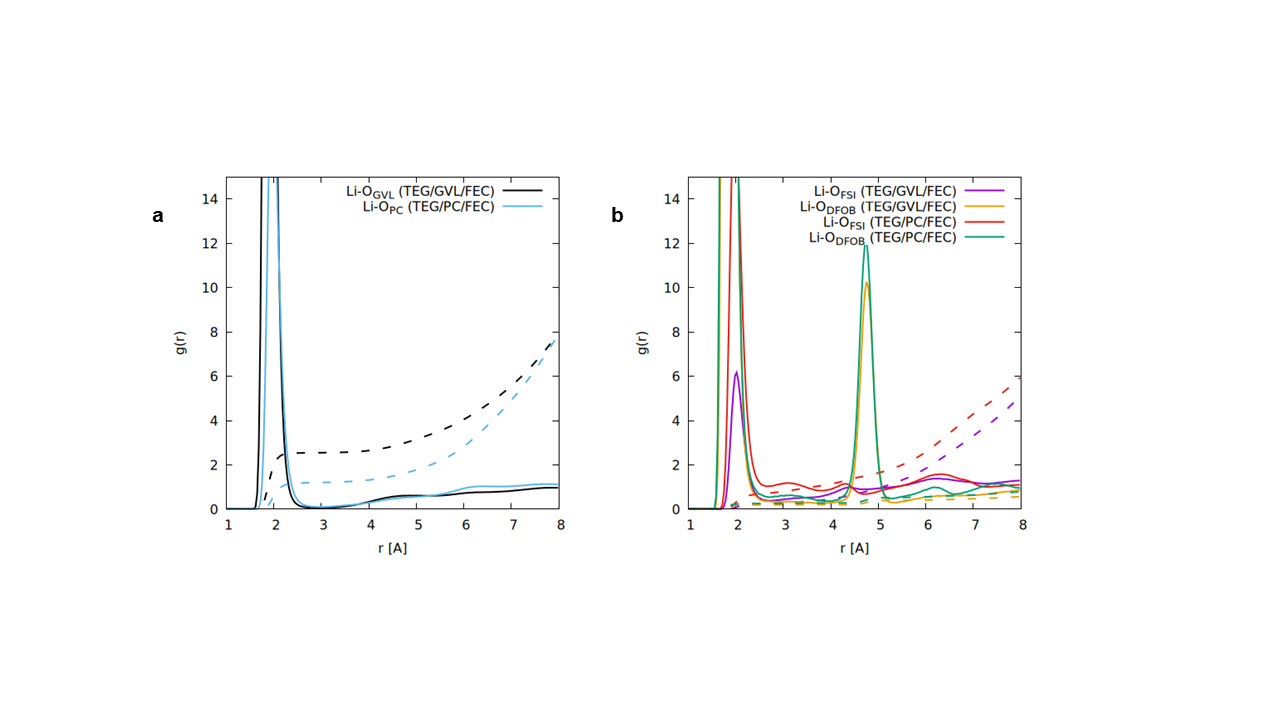


**Figure S1.** Radial distribution functions (solid lines) and their running integrals (dashed lines) for the two selected electrolytes: TEG/GVL/FEC and TEG/PC/FEC. **a)** Lithium-solvent and **b)** lithium-anion radial distribution functions.


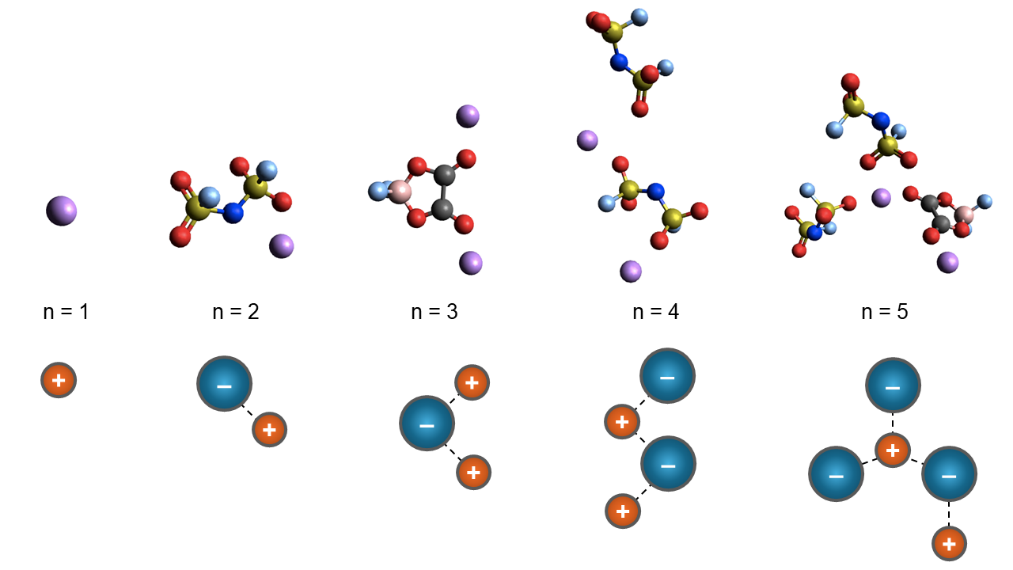


**Figure S2.** Illustrative representation of ionic structures resulting from the cluster analysis of the molecular dynamics simulation. A cluster of order n corresponds to a network of n ions, of any charge sign, interacting to within a distance determined by the first peak in the radial distribution functions.


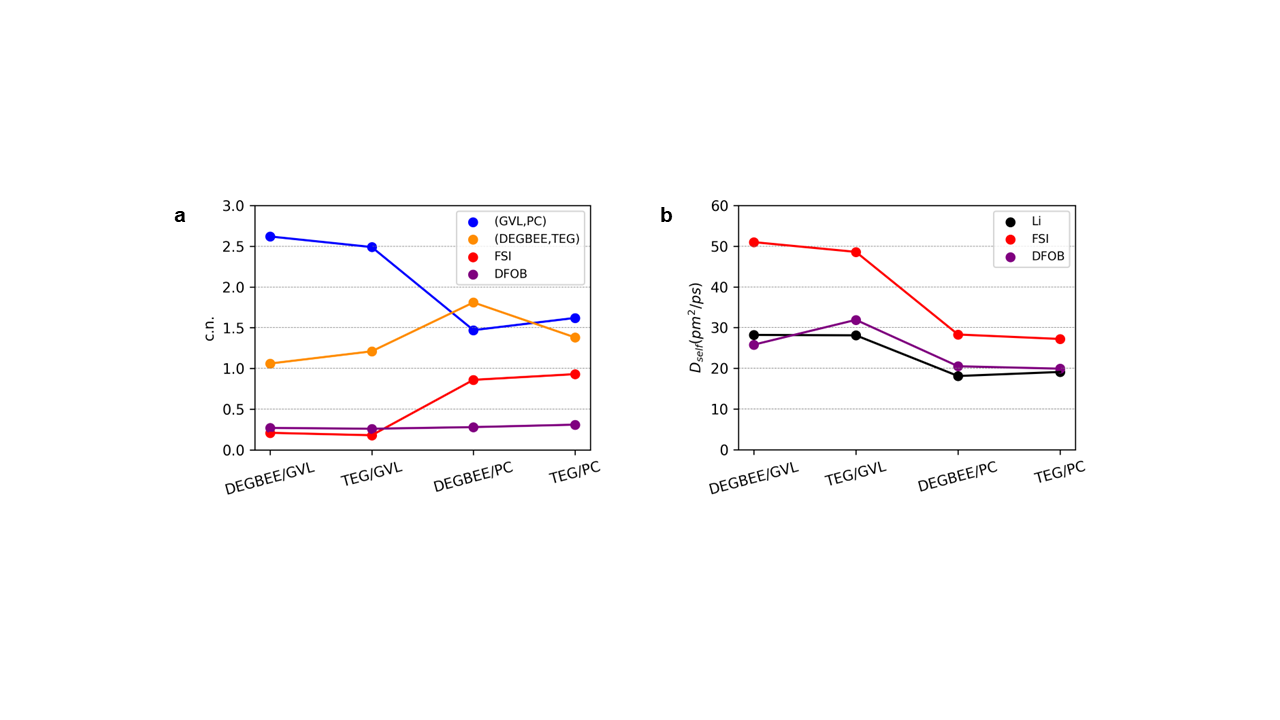


**Figure S3.** Analysis of electrolytes without FEC. **a)** Coordination numbers of Li cations and **b)** self-diffusion coefficients.

**Table S1.** Composition and calculated densities of the simulation cells for electrolytes with FEC.

|  | DEGBEE/GVL/FEC | TEG/GVL/FEC | DEGBEE/PC/FEC | TEG/PC/FEC |
| --- | --- | --- | --- | --- |
| n FSI | 93 | 96 | 103 | 104 |
| n DFOB | 16 | 16 | 17 | 18 |
| n Li | 109 | 112 | 120 | 122 |
| n PC | - | - | 703 | 716 |
| n GVL | 651 | 664 | - | - |
| n TEG | - | 140 |  | 152 |
| n DEGBEE | 147 | - | 162 | - |
| n FEC | 118 | 119 | 130 | 132 |
| density | 1.15 g cm^-3^ | 1.17 g cm^-3^ | 1.23 g cm^-3^ | 1.25 g cm^-3^ |

**Table S2.** Composition and calculated densities of simulation cells for electrolytes without FEC.

|  | DEGBEE/GVL | TEG/GVL | DEGBEE/PC | TEG/PC |
| --- | --- | --- | --- | --- |
| n FSI | 93 | 95 | 103 | 104 |
| n DFOB | 16 | 16 | 17 | 18 |
| n Li | 109 | 111 | 120 | 122 |
| n PC | - | - | 703 | 716 |
| n GVL | 651 | 662 | - | - |
| n TEG | - | 138 | - | 152 |
| n DEGBEE | 147 | - | 162 | - |
| density | 1.14 g cm^-3^ | 1.15 g cm^-3^ | 1.22 g cm^-3^ | 1.23 g cm^-3^ |

**Section 2.** *Electrochemical characterization of the electrolytes*


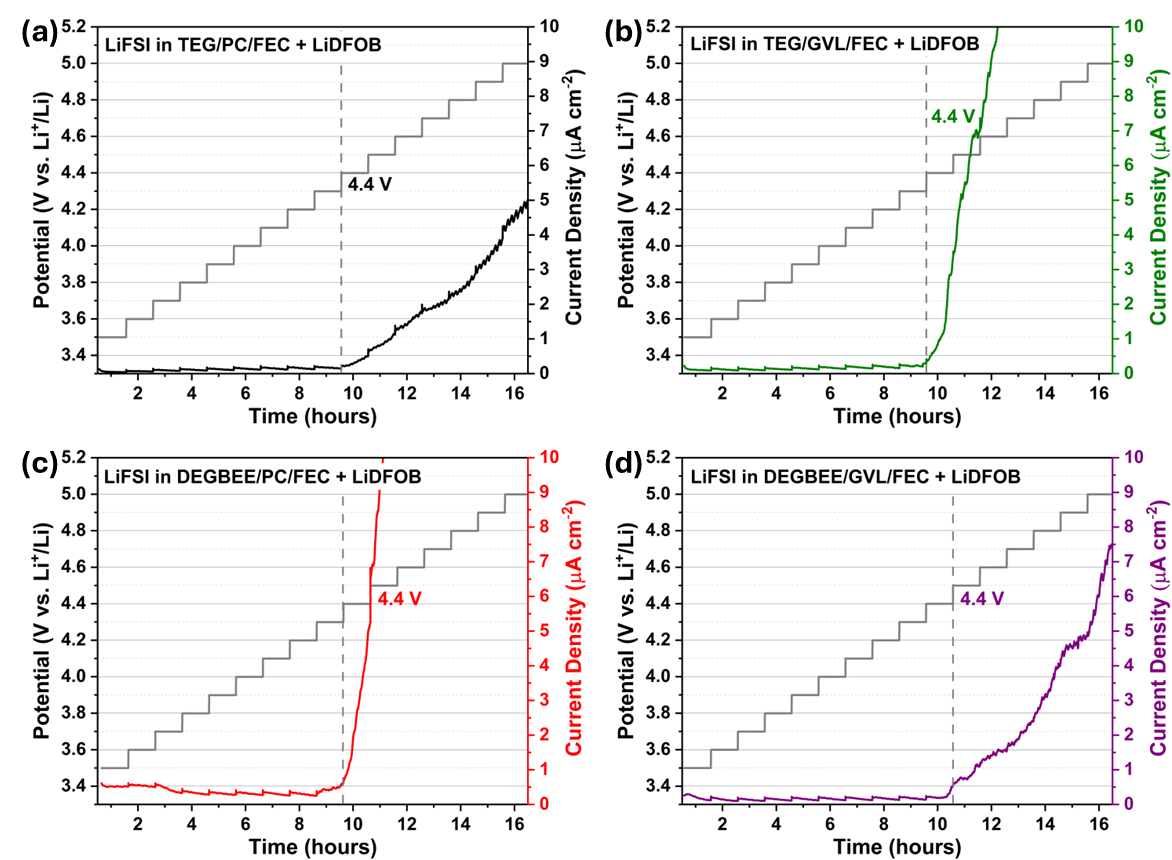


**Figure S4.** Chronoamperometric measurements carried out using an uncoated aluminum current collector against metallic lithium for: **a)** TEG/PC, **b)** TEG/GVL, **c)** DEGBEE/PC, and **d)** DEGBEE/GVL electrolytes.


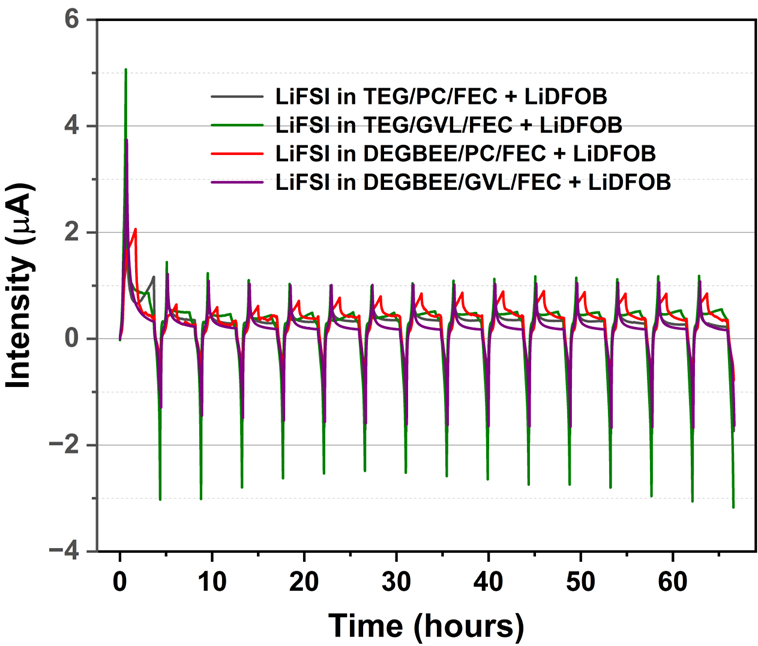


**Figure S5.** Anodic dissolution tests conducted for noted electrolytes in a half-cell configuration consisting of a pristine Al disc as the working electrode and Li metal discs as counter and reference electrodes. Current was registered while the potential of the Al electrode was scanned between 3.0 – 4.3 V vs. Li^+^/Li and held for 3 h at the upper potential limit for each cycle.

**Section 3.** *NMC92 half-cells*

**Table S3.** Detailed cell compositions of the systems explored in this study.

| **Electrochemical studies** | **Cell type** | **Geometry electrodes** | **Working electrode** | **Counter**  **electrode** | **Separator** |
| --- | --- | --- | --- | --- | --- |
| NMC  **half-cell** | Swagelok | Discs  (1.131 cm^2^) | NMC92  (5.4 mg cm^-2^) | Metallic Li | Whatman GF/D glass microfiber |
| Graphite  **half-cell** | Swagelok | Discs  (1.131 cm^2^) | Commercial graphite  (4.7 mg cm^-2^) | Metallic Li | Whatman GF/D glass microfiber |
| LIB  **full-cell** | Swagelok | Discs  (1.131 cm^2^) | NMC92  (5.4 mg cm^-2^) | Commercial graphite  (4.7 mg cm^-2^) | Whatman GF/D glass microfiber |
| LIB  **full-cell** | Pouch cell | Sheets  (10 cm × 6.1 cm and 9.8 cm × 5.9 cm) | Commercial NMC811  (16.3 mg cm^-2^) | Commercial graphite  (9.88 mg cm^-2^) | Celgard H2010+CSP |


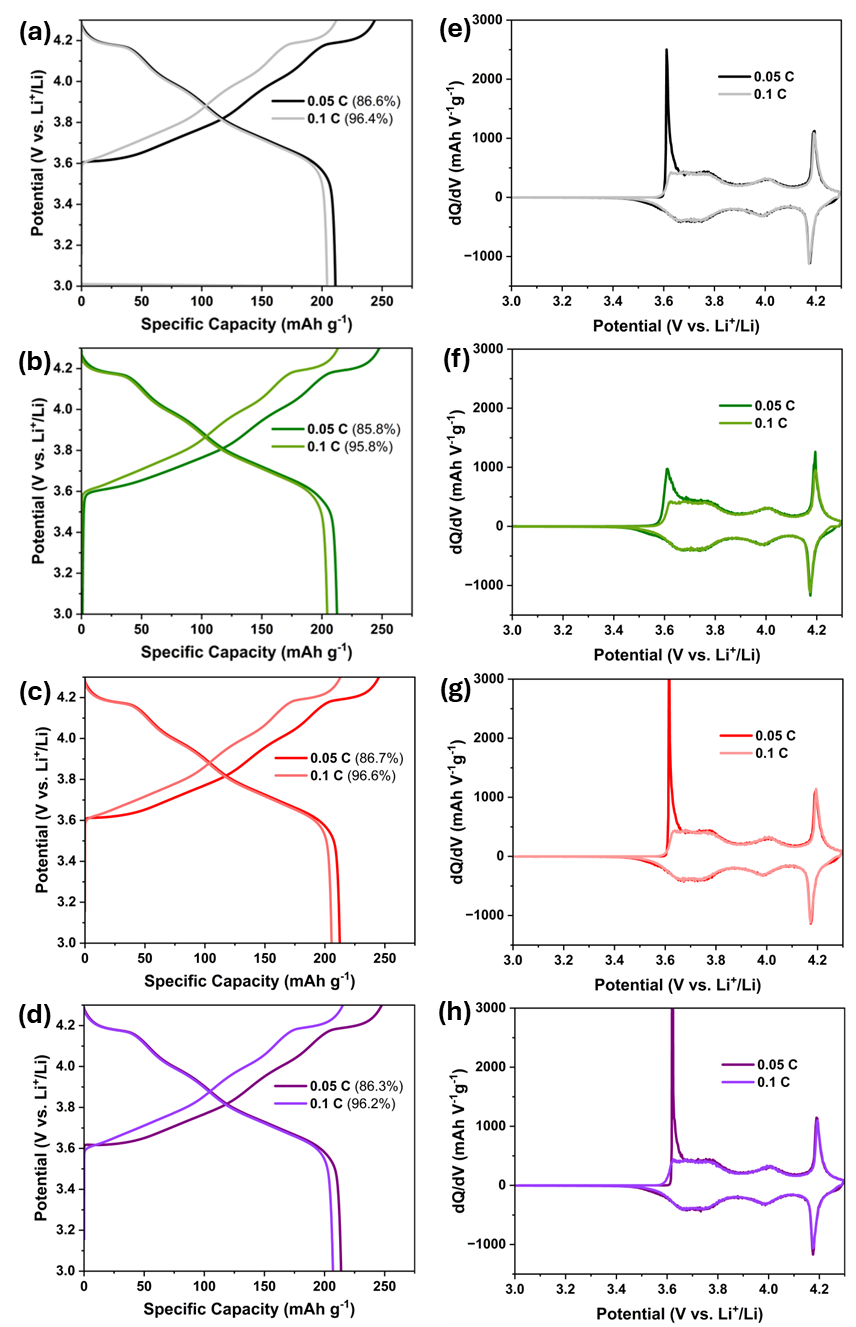


**Figure S6.** First galvanostatic charge/discharge cycles of NMC92 electrodes in half-cell configuration versus metallic lithium using **a)** TEG/PC, **b)** TEG/GVL, **c)** DEGBEE/PC, and **d)** DEGBEE/GVL formulations. **e-h)** Corresponding differential capacity curves.


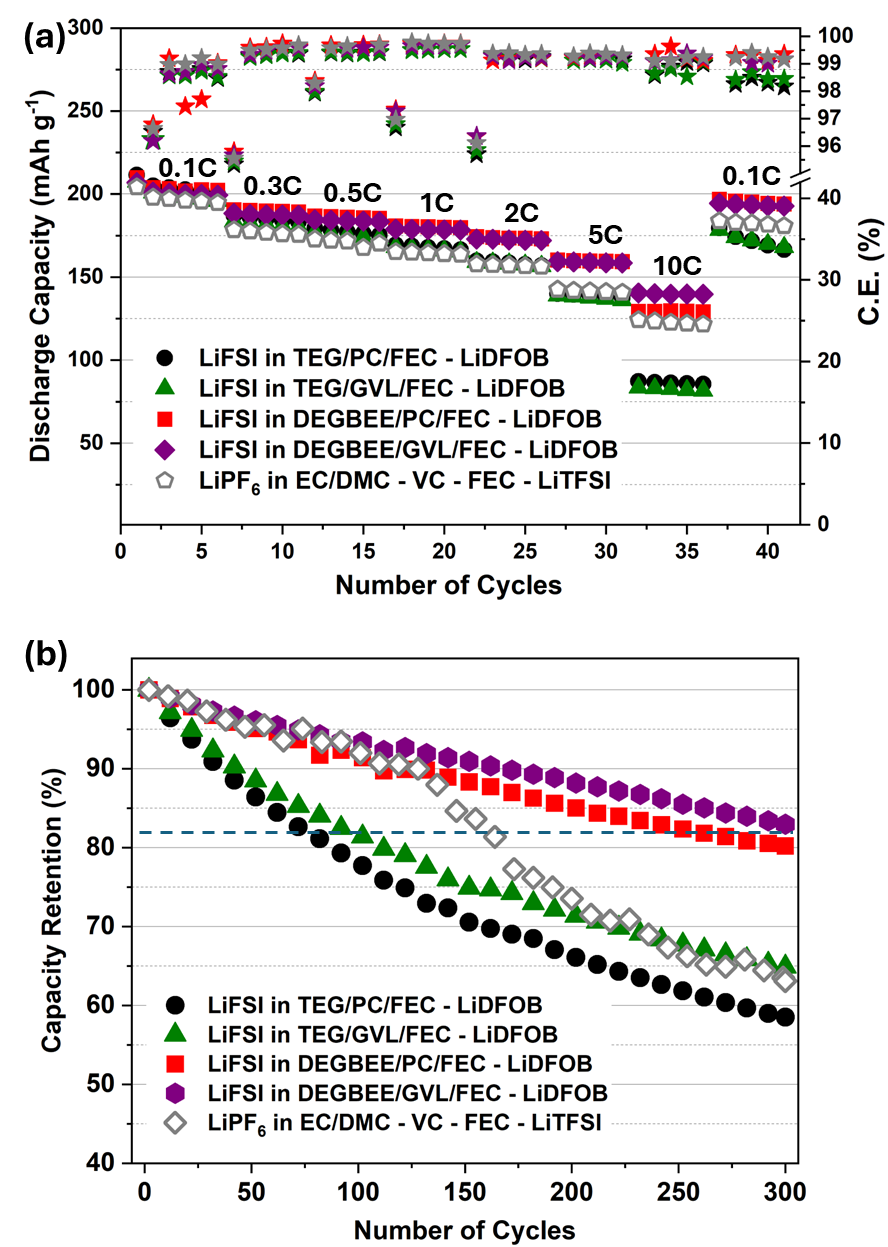


**Figure S7.** **a)** Rate capability tests and **b)** long-term cycling capacity retention at 1 C for NMC92 electrodes in half-cell configuration versus metallic lithium for noted formulations.

**Section 4.** *Ex-situ characterization of cycled NMC92 electrodes*


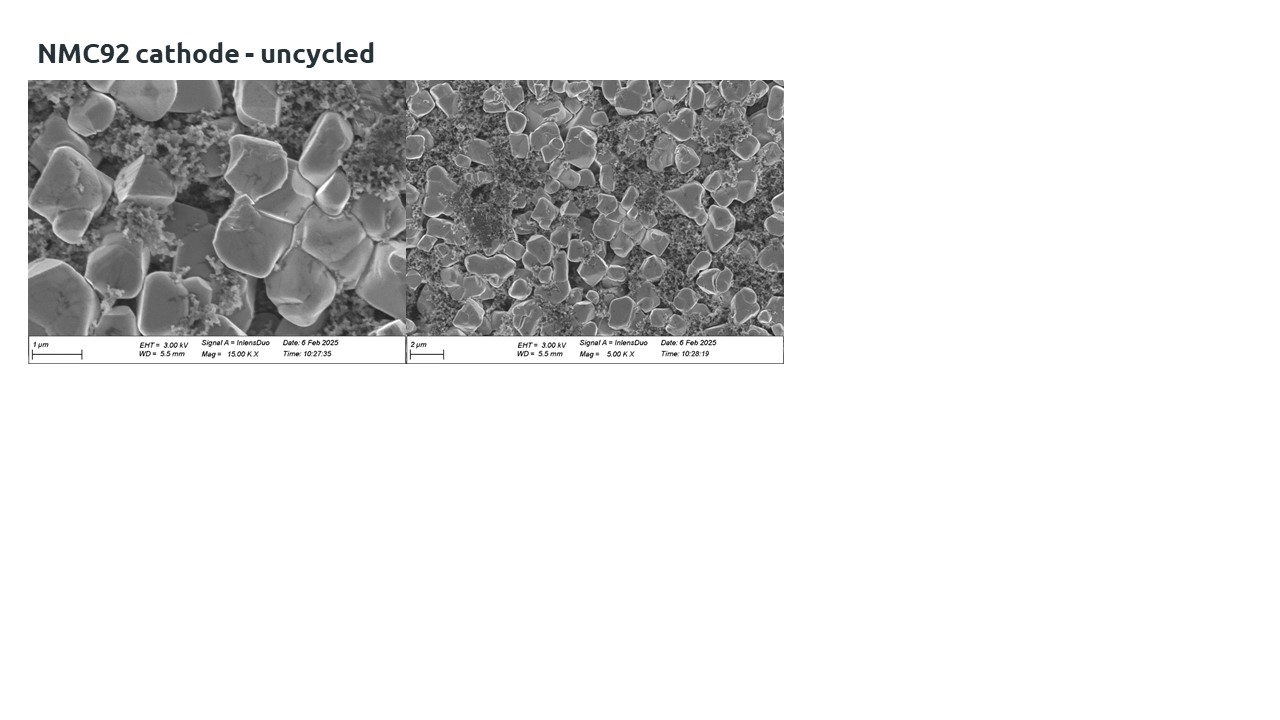


**Figure S8.** SEM images of the uncycled NMC92 cathode.


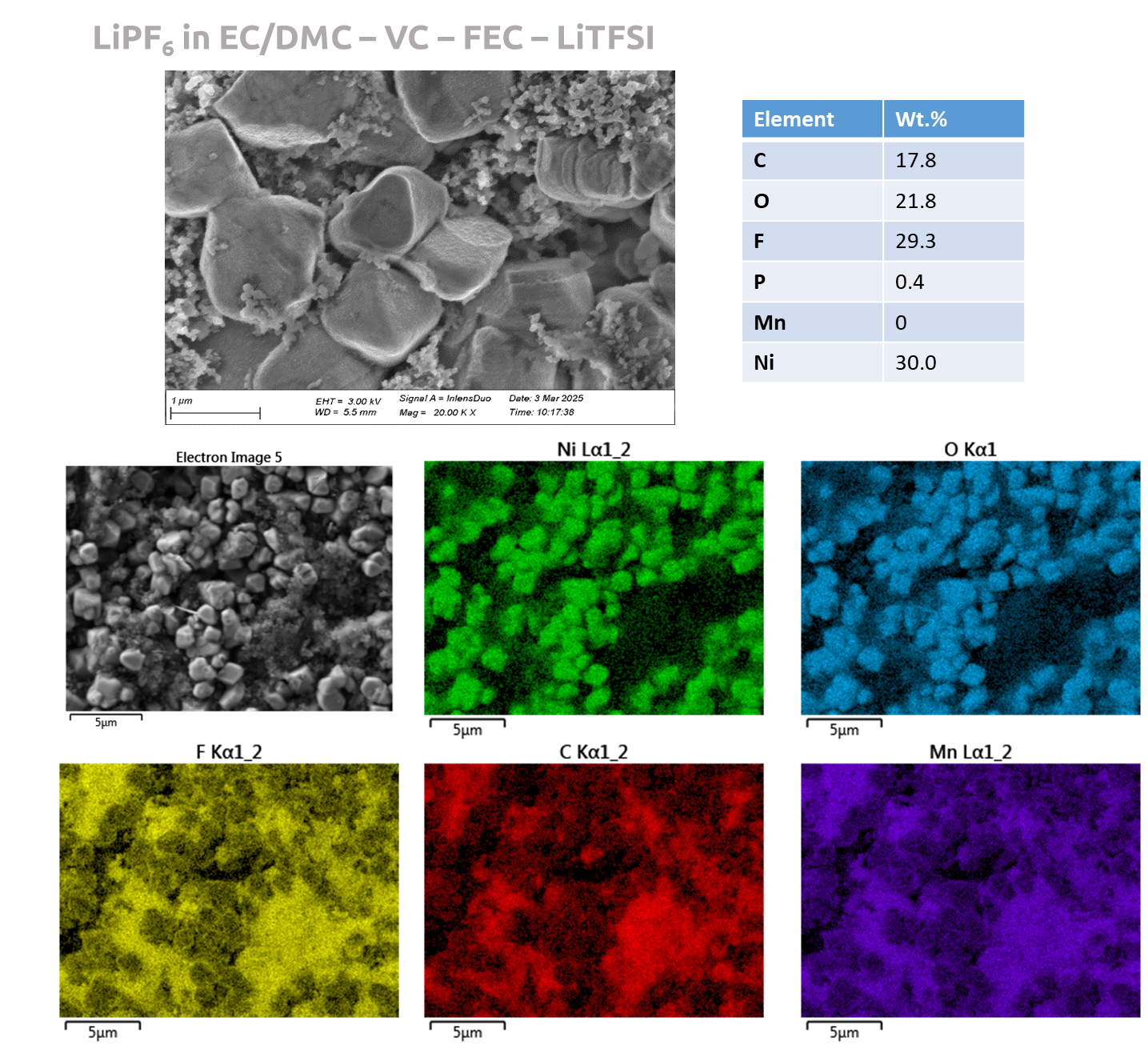


**Figure S9.** SEM image of the NMC92 cathode cycled in the LiPF_6_-based electrolyte including EDX elemental maps and composition table in wt.%.


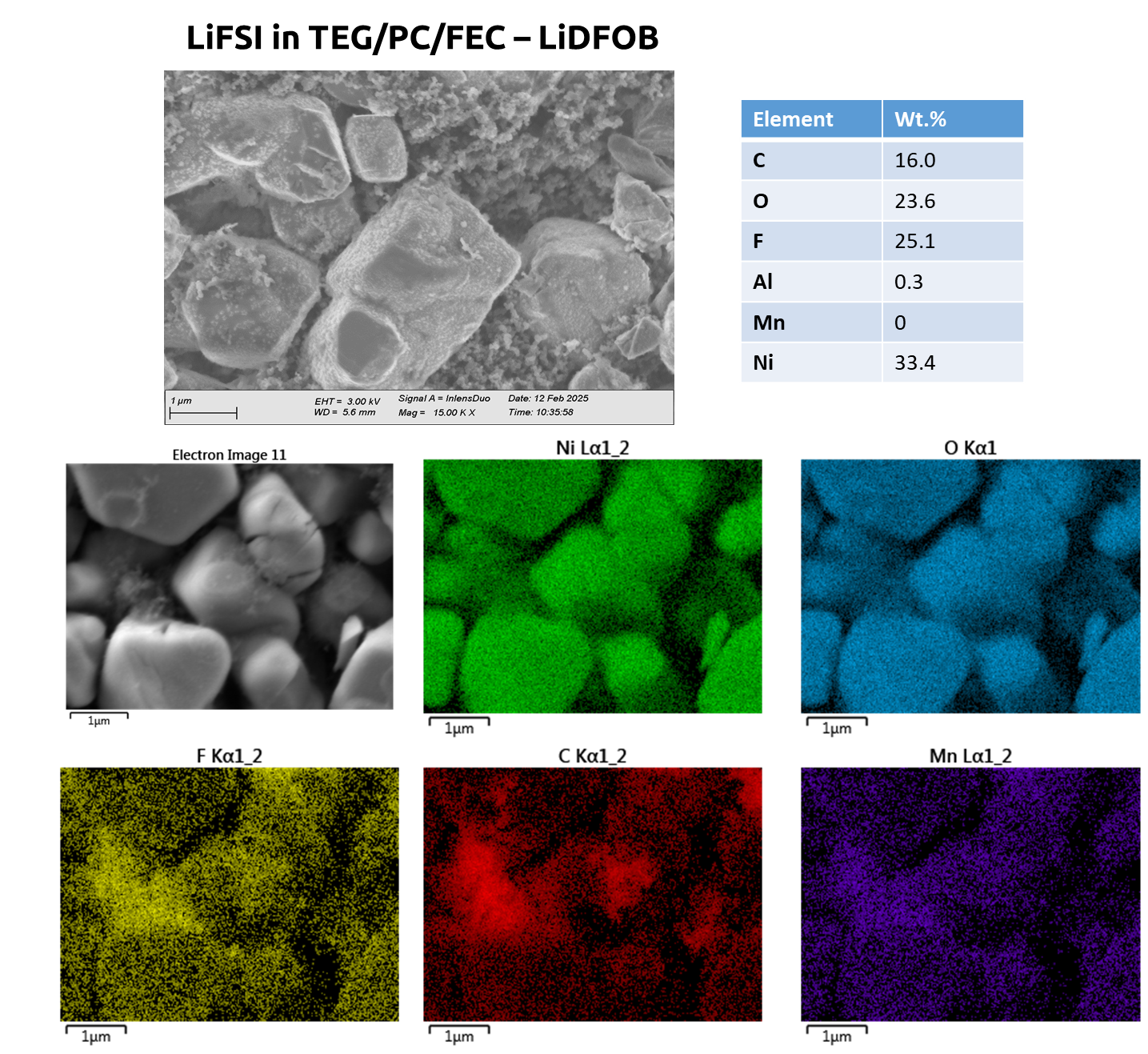


**Figure S10.** SEM image of the NMC92 cathode cycled in the LiFSI-based electrolyte with TEG/PC solvent including EDX elemental maps and composition table in wt.%.


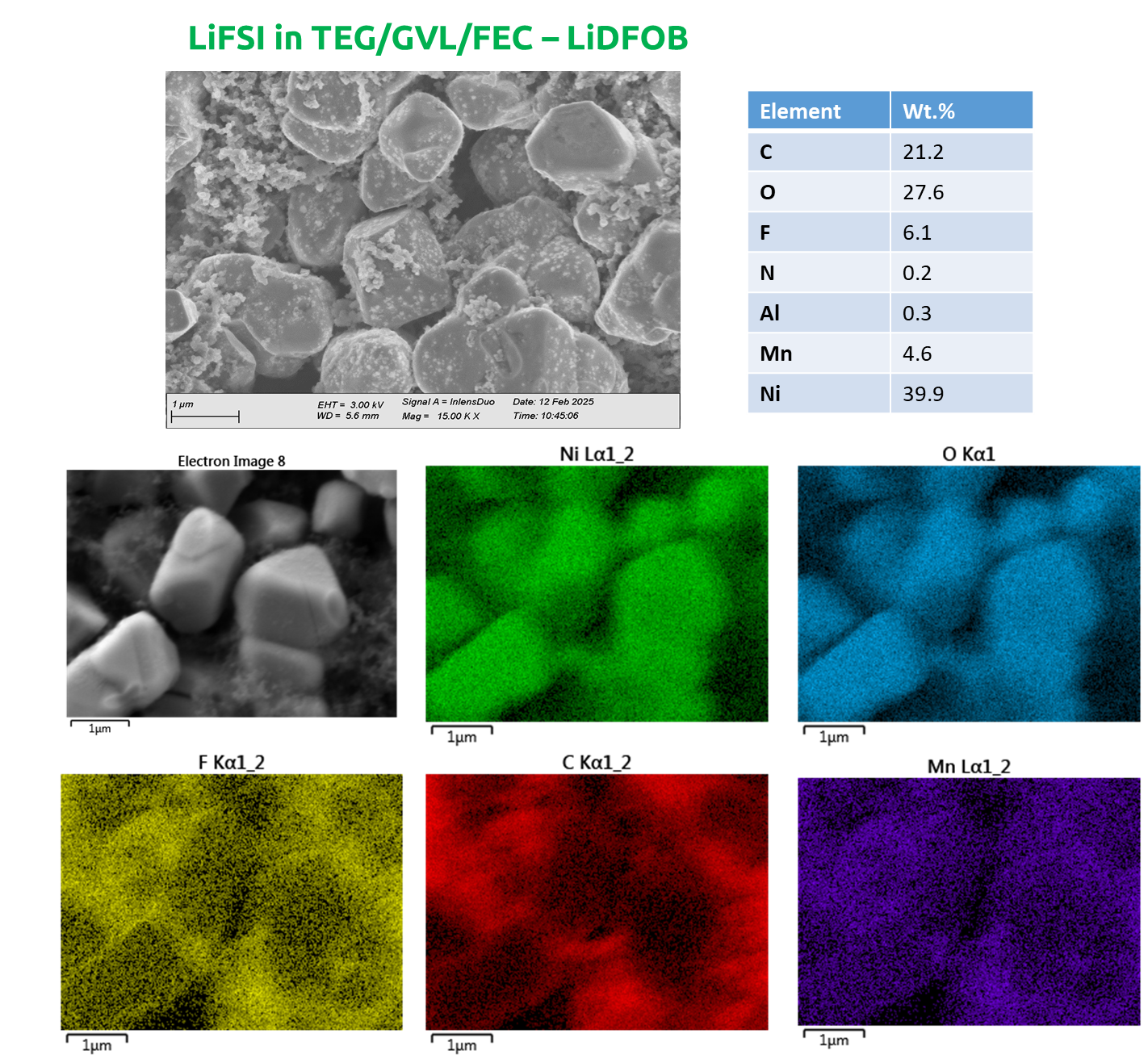


**Figure S11.** SEM image of the NMC92 cathode cycled in the LiFSI-based electrolyte with TEG/GVL solvent including EDX elemental maps and composition table in wt.%.


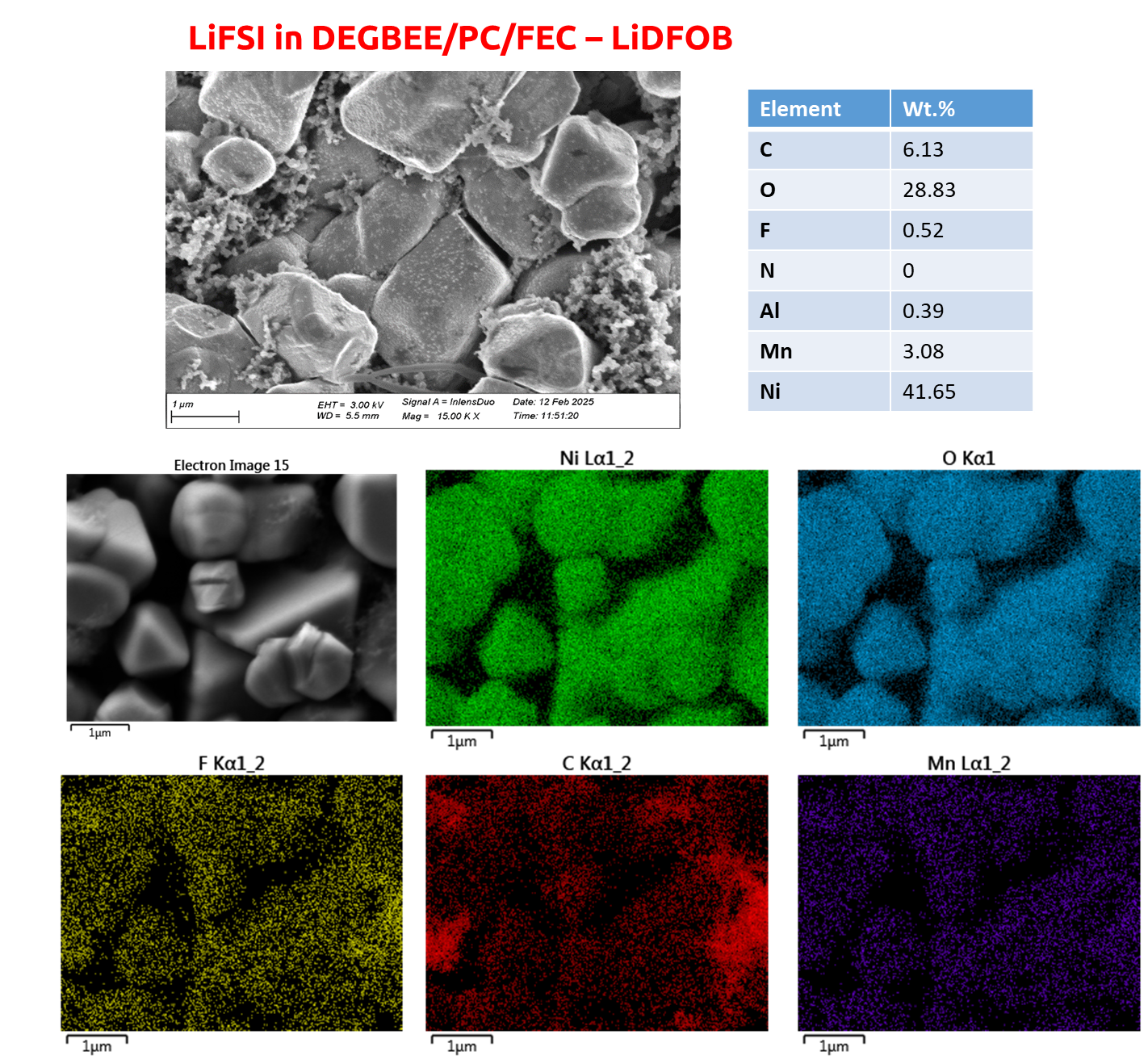


**Figure S12.** SEM image of the NMC92 cathode cycled in the LiFSI-based electrolyte with DEGBEE/PC solvent including EDX elemental maps and composition table in wt.%.


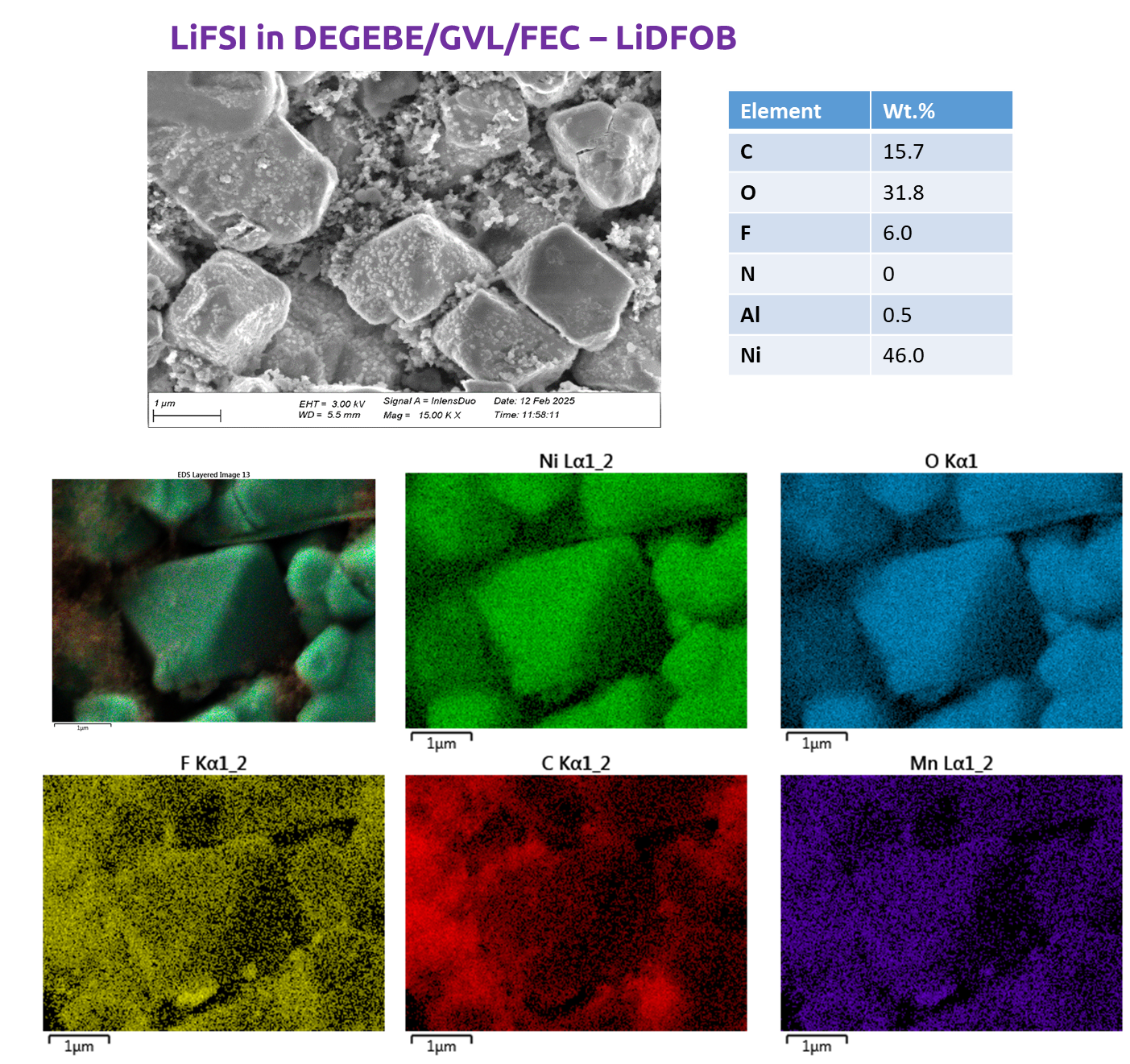


**Figure S13.** SEM image of the NMC92 cathode cycled in the LiFSI-based electrolyte with DEGBEE/GVL solvent including EDX elemental maps and composition table in wt.%.


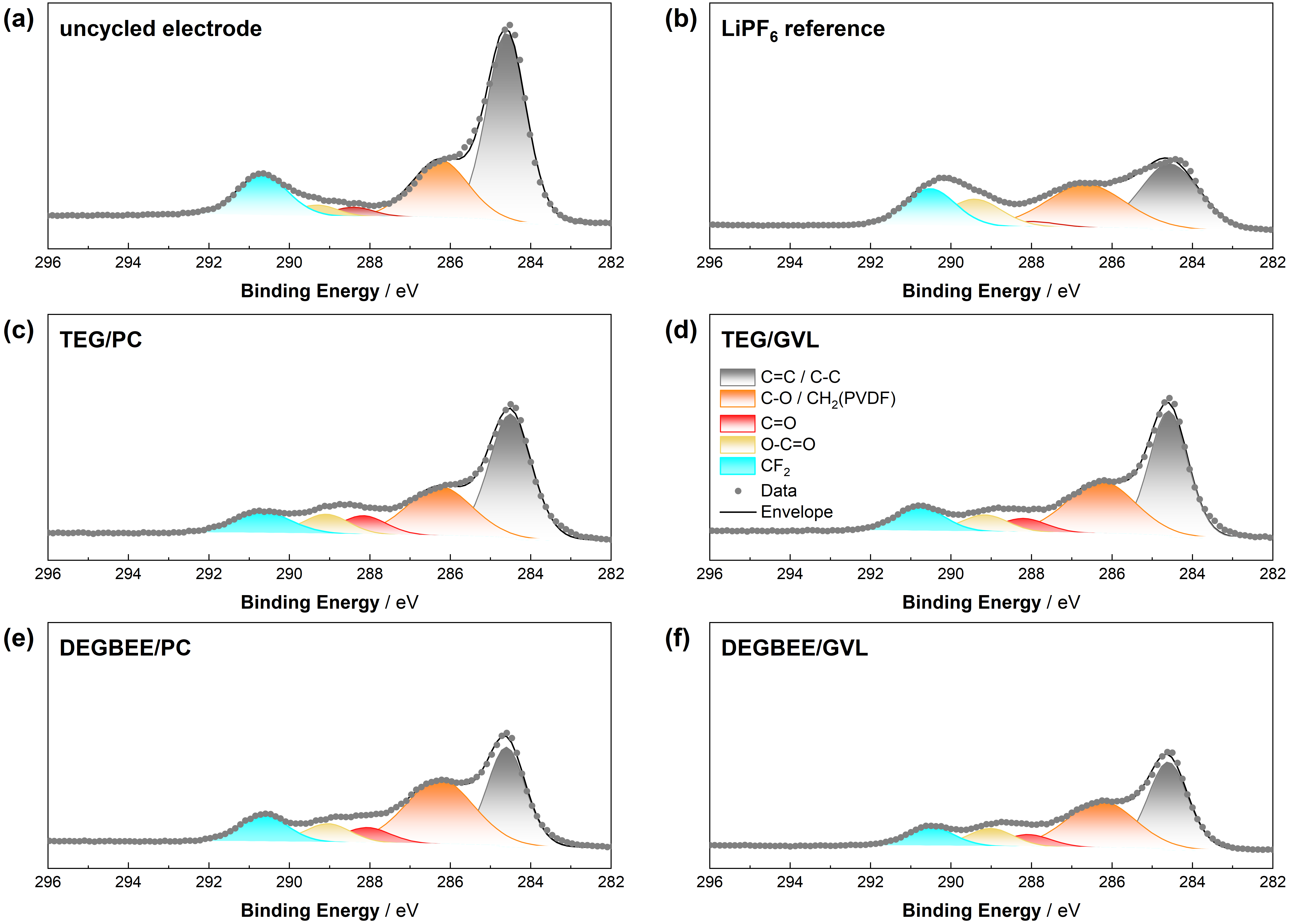


**Figure S14.** C1s region of the XPS detail spectra of the NMC92 cathodes recorded for **(a)** uncycled, or after 300 cycles at 1C in **(b)** LiPF_6_ in DMC/EC, **(c)** LiFSI in TEG/PC, **(d)** LiFSI in TEG/GVL, **(e)** LiFSI in DEGBEE/PC, and **(f)** LiFSI in DEGBEE/GVL.


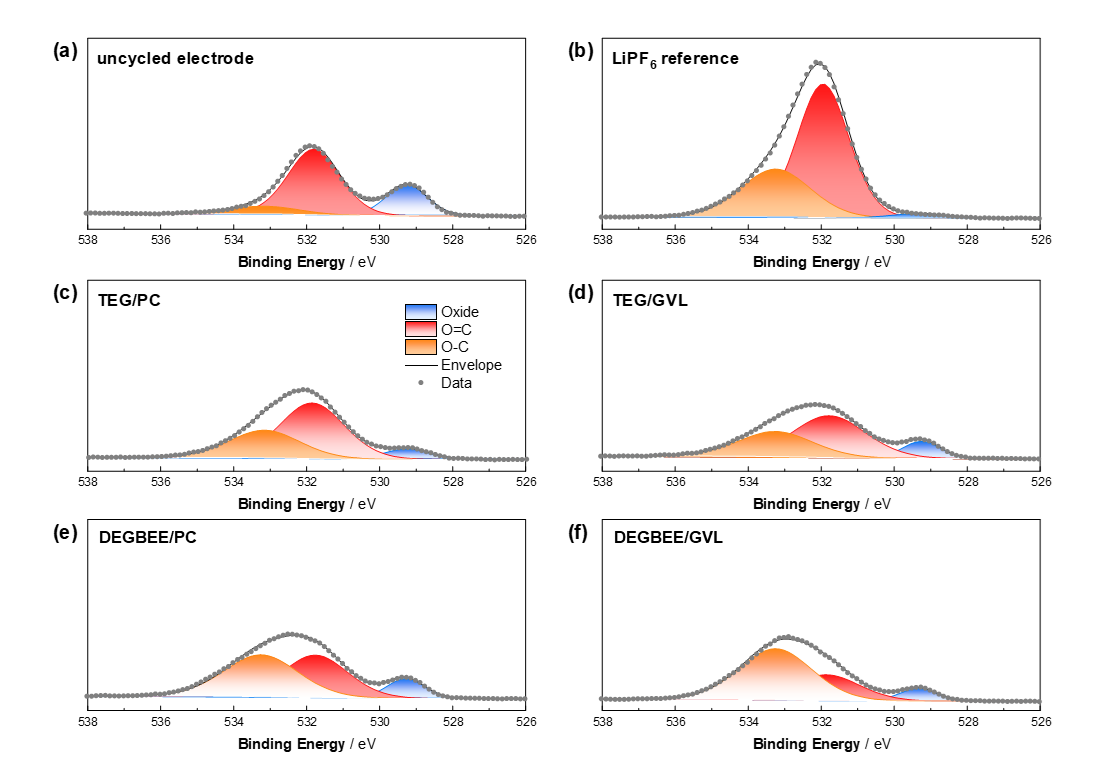


**Figure S15.** O1s region of the XPS detail spectra of the NMC92 cathodes recorded for **(a)** uncycled, or after 300 cycles at 1C in **(b)** LiPF_6_ in DMC/EC, **(c)** LiFSI in TEG/PC, **(d)** LiFSI in TEG/GVL, **(e)** LiFSI in DEGBEE/PC, and **(f)** LiFSI in DEGBEE/GVL.


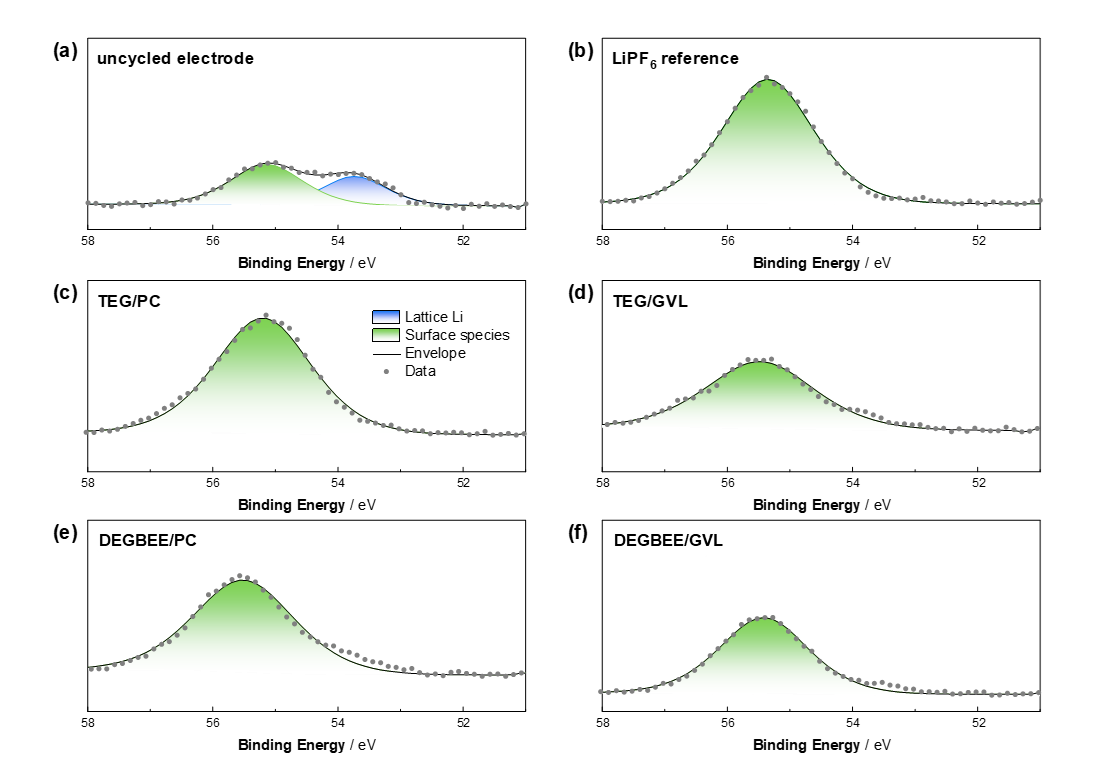


**Figure S16.** L1s region of the XPS detail spectra of the NMC92 cathodes recorded for **(a)** uncycled, or after 300 cycles at 1C in **(b)** LiPF_6_ in DMC/EC, **(c)** LiFSI in TEG/PC, **(d)** LiFSI in TEG/GVL, **(e)** LiFSI in DEGBEE/PC, and **(f)** LiFSI in DEGBEE/GVL.


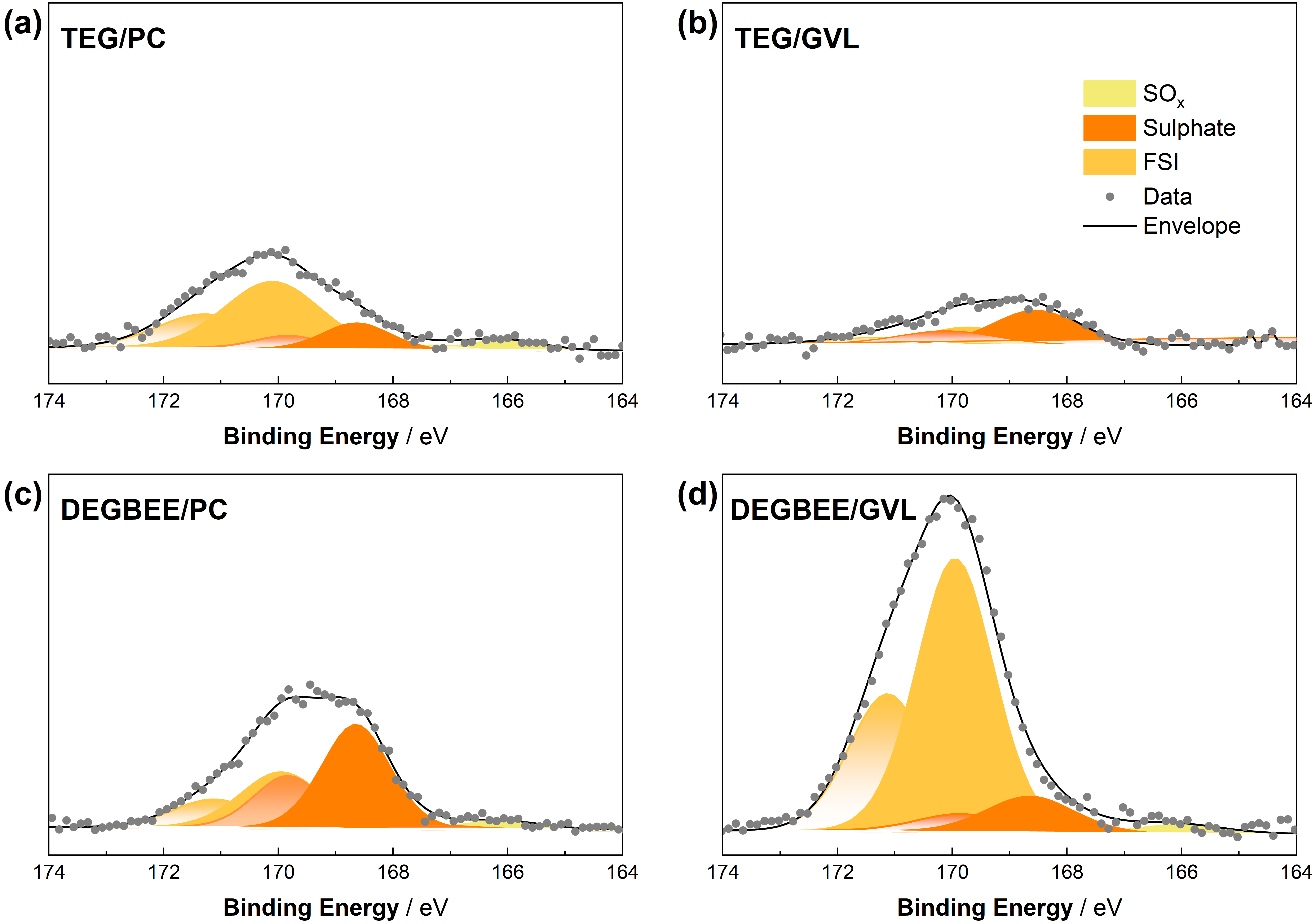


**Figure S17.** S2p region of the XPS detail spectra of the NMC92 cathodes recorded after 300 cycles at 1C in **(a)** LiFSI in TEG/PC, **(b)** LiFSI in TEG/GVL, **(c)** LiFSI in DEGBEE/PC, and **(d)** LiFSI in DEGBEE/GVL.

**Table S4.** Elemental composition of the NMC92 electrode surface determined by quantitative analysis of the XPS spectra (NMC transition metals were excluded from analysis due to their small concentrations).

| At. % | **Carbon** | | | | | **Oxygen** | | | **Fluorine** | | **Lithium** | | Sulphur | Boron |
| --- | --- | --- | --- | --- | --- | --- | --- | --- | --- | --- | --- | --- | --- | --- |
|  | **C-C** | **C-O** | **C=O** | **O-C=O** | **C-F** | **M-O** | **O-C** | **O=C** | **M-F** | **C/P-F** | **Li-O** | **Li-X** | **Total** | **Total** |
| **Uncycled** | *29.4* | *12.8* | *1.5* | *1.8* | *8.2* | *5.3* | *14.2* | *2.2* | *0.7* | *15.4* | *2.9* | *4.6* |  |  |
| **total** |  |  |  |  | **53.8** |  |  | **21.7** |  | **16.1** |  | **7.5** | **0.2** | **0** |
| **LiPF_6_** | *12.8* | *11.4* | *1* | *4.9* | *6.2* | *0.9* | *24.1* | *11.6* | *3.8* | *7.6* | *0* | *15* |  |  |
| **total** |  |  |  |  | **36.3** |  |  | **36.6** |  | **11.4** |  | **15** | **0** | **0** |
| **TEG/PC** | *17.5* | *10.6* | *2.9* | *3* | *4.2* | *1.7* | *12.5* | *6.8* | *16.4* | *8* | *0* | *14.8* |  |  |
| **total** |  |  |  |  | **38.2** |  |  | **21** |  | **24.4** |  | **14.8** | **0.3** | **1** |
| **TEG/GVL** | *19* | *14.1* | *2.5* | *3.1* | *4.8* | *2.7* | *12.5* | *7.7* | *10.9* | *9.9* | *0* | *11.5* |  |  |
| **total** |  |  |  |  | **43.6** |  |  | **22.9** |  | **20.8** |  | **11.5** | **0.2** | **0.8** |
| **DEGBEE/PC** | *13.5* | *15.7* | *2.5* | *3.1* | *4.7* | *2.8* | *10.3* | *11.6* | *12.4* | *8.6* | *0* | *13.3* |  |  |
| **total** |  |  |  |  | **39.6** |  |  | **24.7** |  | **21** |  | **13.3** | **0.5** | **0.5** |
| **DEGBEE/GVL** | *14.9* | *13.7* | *2.4* | *3.6* | *4* | *2.2* | *7.7* | *16.8* | *12.3* | *8.2* | *0* | *11.6* |  |  |
| **total** |  |  |  |  | **38.6** |  |  | **26.7** |  | **20.5** |  | **11.6** | **1.1** | **0.6** |

**Section 5.** *Graphite half-cell characterization*


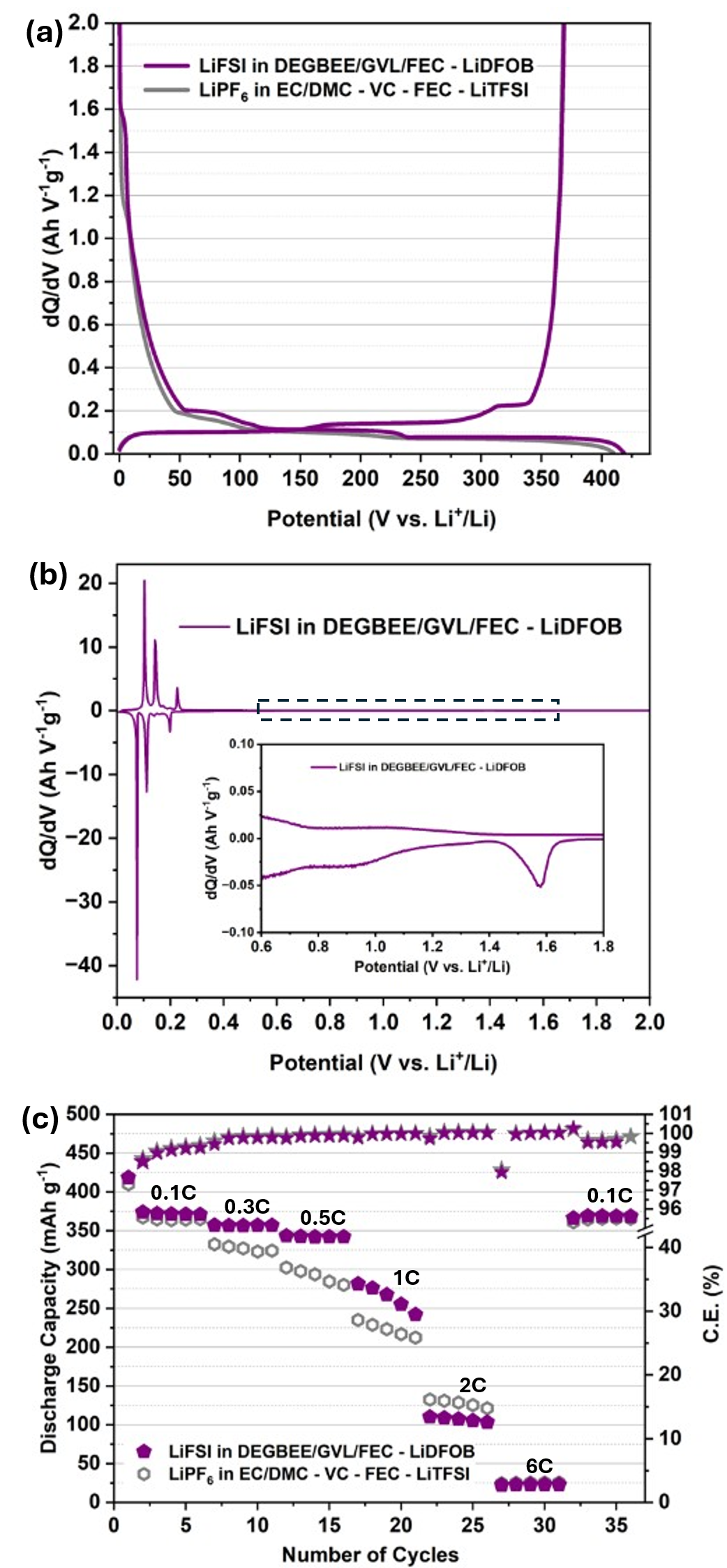


**Figure S18.** **a)** First cycle galvanostatic charge/discharge profiles of the graphite electrodes in half-cell configuration versus metallic lithium using the DEGBEE/GVL and LiPF_6_-based electrolytes, **b)** corresponding differential capacity curves for the DEGBEE/GVL electrolyte, and **c)** rate capability tests for the DEGBEE/GVL and LiPF_6_-based electrolytes.

**Section 6.** *Lithium-ion battery pouch cell testing*


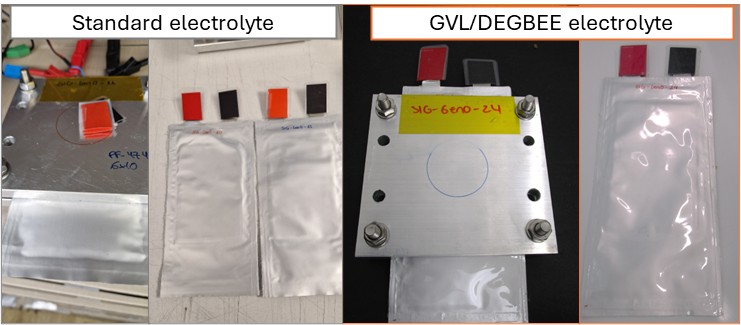


**Figure 19.** Digital photograph of the assembled lithium-ion battery pouch cells consisting of graphite negative electrodes and NMC_811_ positive electrodes after the initial formation cycle at 0.05 C.


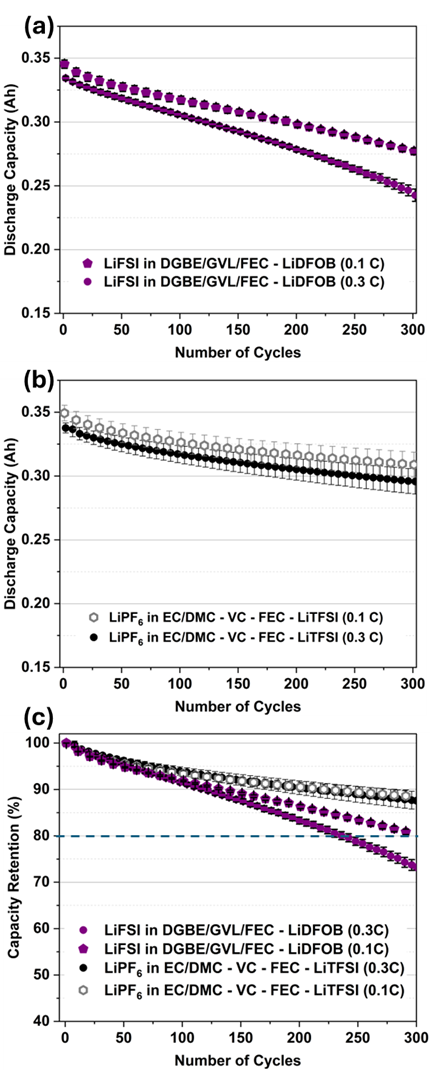


**Figure S20**. **a)** Galvanostatic cycling for DEGBEE/GVL and **b)** LiPF_6_-based electrolyte in a LIB pouch-cell consisting of graphite negative electrodes and NMC_811_ positive electrodes, showing the capacity for both the cycles performed at 0.3 C and 0.1 C. **c)** Corresponding capacity retention for both current densities. Error bars correspond to the standard deviation obtained from three independent measurements.


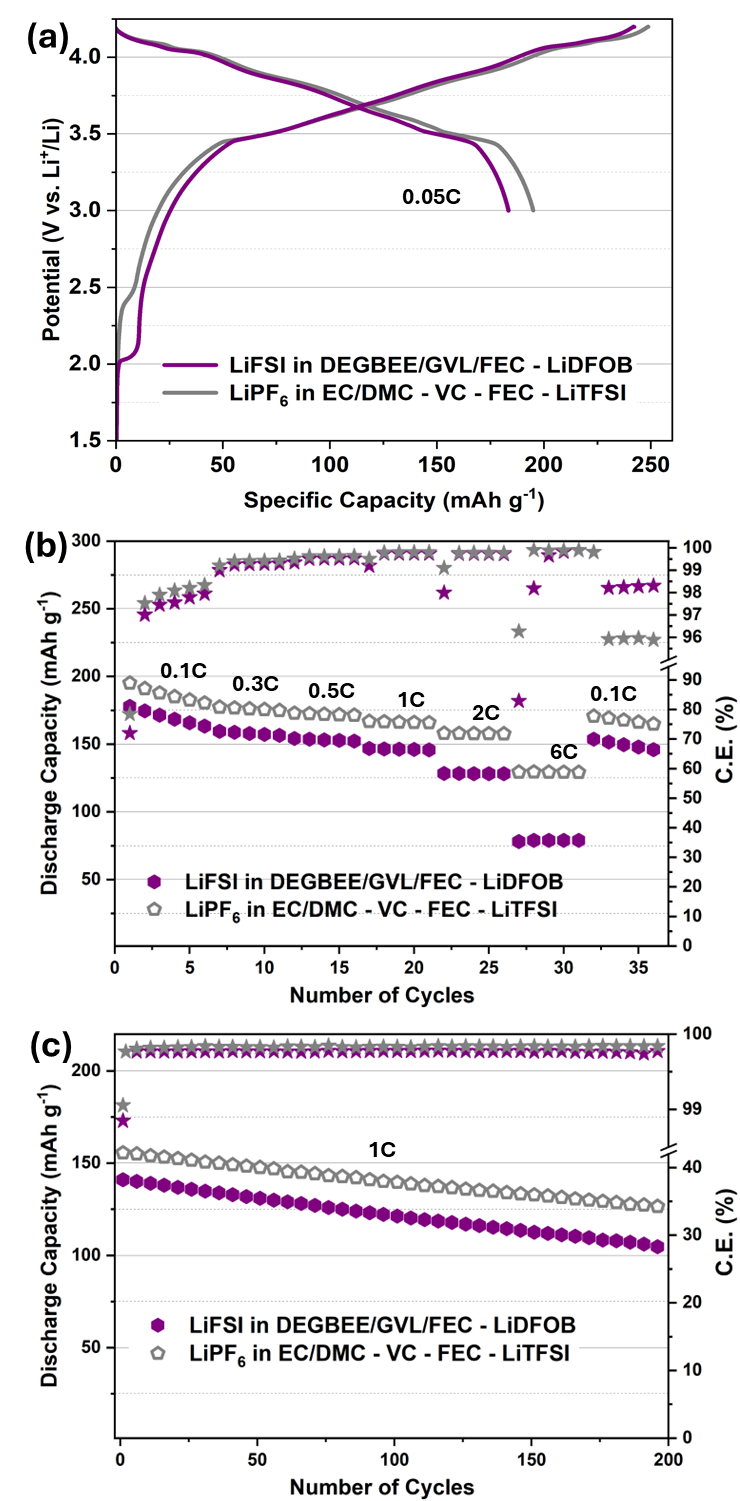


**Figure S21.** Electrochemical characterization of LIB lab-scale cells consisting of NMC92 and graphite, including: **a)** first cycle galvanostatic charge/discharge profiles at 0.05 C, **b)** rate capability tests, and **c)** long-term cycling at 1 C for the DEGBEE/GVL and LiPF_6_-based electrolytes.


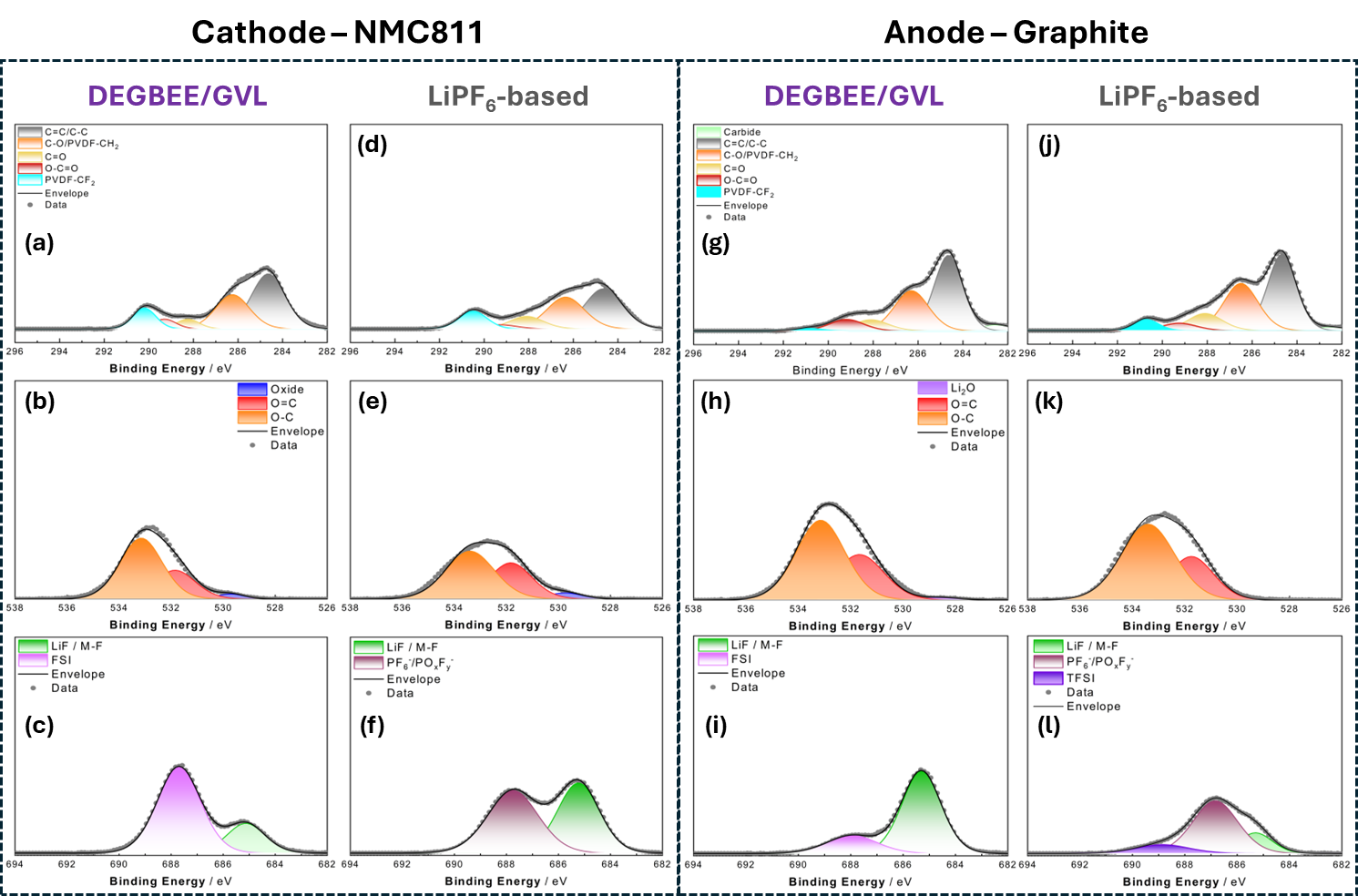


**Figure S22.** XPS detail spectra of pouch-cell electrodes collected after reaching 80% of their initial capacity. NMC811 cathode cycled with LiFSI in DEGBEE/GVL electrolyte showing the **(a)** C1s, **(b)** O1s, and **(c)** F1s regions, and **(d-f)** the corresponding spectra for the NMC811 cathode cycled with the LiPF_6_ in EC/DMC electrolyte. Graphite anode cycled with LiFSI in DEGBEE/GVL electrolyte showing the **(g)** C1s, **(h)** O1s, and **(i)** F1s regions, and **(j-l)** the corresponding spectra for the graphite anode cycled with the LiPF_6_ in EC/DMC electrolyte.

**Table S5.** Elemental composition of the NMC811 cathode and graphite anode surfaces determined by quantitative analysis of the XPS spectra.

| At. % | **Carbon** | | | | | **Oxygen** | | | | **Fluorine** | | | **Lithium** | | Sulphur | Boron | | Nitrogen | | |
| --- | --- | --- | --- | --- | --- | --- | --- | --- | --- | --- | --- | --- | --- | --- | --- | --- | --- | --- | --- | --- |
|  | **C-C** | **C-O** | **C=O** | **O-C=O** | **C-F** | **Li_2_O** | **M-O** | **O-C** | **O=C** | **M-F** | **PO_x_F_y_^-^** | **C/P-F** | **Li-O** | **Li-X** | **Total** | **B-O** | **B-F** | **Nitride** | **FSI^-^/TFSI^-^** |  |
| **DEGBEE/GVL** (NMC811) | *21.16* | *13.21* | *2.54* | *2.51* | *5.40* |  | *0.8* | *6.76* | *14.94* | *4.91* | *0* | *16.19* | *0* | *7.53* |  | 0.79 | 0.38 | 0 | 1.08 |  |
| **Total** |  |  |  |  | **44.82** |  |  |  | **22.5** |  |  | **21.1** |  | **7.53** | **1.79** |  | **1.17** |  | **1.08** |  |
| **LiPF_6_**  (NMC811) | *15.64* | *12.47* | *4.45* | *1.48* | *5.17* |  | *1.09* | *8.13* | *12.42* | *11.68* | *12.53* | *0* | *0* | *13.62* |  | 0 | 0 | 0 | 0.19 |  |
| **Total** |  |  |  |  | **39.22** |  |  |  | **21.64** |  |  | **24.2** |  | **13.62** | **0.14** |  | **0** |  | **0.19** |  |
| **DEGBEE/GVL** (Graphite) | *18.27* | *11.72* | *3.24* | *3.33* | *0.54* | *0.36* | *0* | *9.75* | *17.13* | *10.51* | *0* | *2.96* | *0* | *15.6* |  | 1.24 | 0.48 | 0.36 | 1.04 |  |
| **Total** |  |  |  |  | **38.58** |  |  |  | **27.24** |  |  | **13.46** |  | **15.6** | **1.99** |  | **1.73** |  | **1.4** |  |
| **LiPF_6_**  (Graphite) | *20.84* | *15.49* | *5.61* | *2.42* | *2.57* | *0* | *0* | *9.5* | *19.96* | *2.51* | *8.66* | *1.67* | *0* | *8.05* |  | 0 | 0 | 0 | 0.24 |  |
| **Total** |  |  |  |  | **47.92** |  |  |  | **29.46** |  |  | **12.84** |  | **8.05** | **0.15** |  | **0** |  | **0.24** |  |
